# Single-cell RNA combined with bulk RNA analysis to explore oxidative stress and energy metabolism factors and found a new prostate cancer oncogene *MXRA8*

**DOI:** 10.18632/aging.205599

**Published:** 2024-03-04

**Authors:** Miao Miao, Yan Song, Mingyue Jin, Yang Du, Peng Xin, Yuanjun Jiang, Hao Zhang

**Affiliations:** 1 Department of Urology, The First Hospital of China Medical University, Shenyang 110001, China; 2 Operating Room, The First Hospital of China Medical University, Shenyang 110001, China; 3 Department of Endocrinology, Shenzhen University General Hospital, Shenzhen, Guangdong, China

**Keywords:** oxidative stress, prostate cancer, prognostic, *MXRA8*, energy metabolism

## Abstract

Background: Prostate cancer is the most common malignancy among men worldwide, and its diagnosis and treatment are challenging due to its heterogeneity.Methods: Integrating single-cell RNA sequencing (scRNA-seq) and bulk RNA-seq data, we identified two molecular subtypes of prostate cancer based on dysregulated genes involved in oxidative stress and energy metabolism. We constructed a risk score model (OMR) using common differentially expressed genes, which effectively evaluated prostate cancer prognosis.

Results: Our analysis demonstrated a significant correlation between the risk score model and various factors, including tumor immune microenvironment, genomic variations, chemotherapy resistance, and immune response. Notably, patients with low-risk scores exhibited increased sensitivity to chemotherapy and immunotherapy compared to those with high-risk scores, indicating the model’s potential to predict patient response to treatment. Additionally, our investigation of *MXRA8* in prostate cancer showed significant upregulation of this gene in the disease as confirmed by PCR and immunohistochemistry. Functional assays including CCK-8, transwell, plate cloning, and ROS generation assay demonstrated that depletion of *MXRA8* reduced the proliferative, invasive, migratory capabilities of PC-3 cells, as well as their ROS generation capacity.

Conclusions: Our study highlights the potential of oxidative stress and energy metabolism-related genes as prognostic markers and therapeutic targets in prostate cancer. The integration of scRNA-seq and bulk RNA-seq data enables a better understanding of prostate cancer heterogeneity and promotes personalized treatment development. Additionally, we identified a novel oncogene *MXRA8* in prostate cancer.

## INTRODUCTION

Prostate cancer is a leading cause of cancer-related deaths among men worldwide, with approximately 1.4 million cases and over 375,000 deaths annually [1, 2]. It is a complex disease that displays significant heterogeneity in terms of clinical presentation, molecular characteristics, and treatment response [3, 4]. While early detection and treatment for prostate cancer have improved significantly, patients with advanced prostate cancer still have a relatively low long-term survival rate [5]. An imbalance between the generation of reactive oxygen species (ROS) and antioxidant defense mechanisms is responsible for oxidative stress, which ultimately causes cellular dysfunction and damage [6]. ROS generated during oxidative metabolism can induce DNA damage, genomic instability, and oncogenic signaling, promoting tumor growth and progression [7]. On the other hand, alterations in energy metabolism, such as dysregulated mitochondrial function and altered nutrient uptake, can also impact tumor cell survival and proliferation [8, 9].

Recent technological advancements in scRNA sequencing have allowed for the assessment of intratumoral heterogeneity, which refers to the diversity of cells within a tumor [10]. Intratumoral heterogeneity can drive tumor progression, resistance to therapy, and poor prognosis [11]. Additionally, bulk RNA sequencing enables the identification of differentially expressed genes in cancer tissue compared to normal tissue [12].

Given the impact of oxidative stress and energy metabolism on tumor development and progression, exploring their role in prostate cancer may yield valuable insights into the molecular mechanisms underlying disease pathogenesis and progression. Furthermore, identifying key prognostic factors associated with aggressive phenotypes and poor prognosis can aid in developing personalized treatment strategies and improving patient outcomes. Therefore, we aim to use scRNA and bulk RNA sequencing to investigate the role of oxidative stress and energy metabolism factors in prostate cancer prognosis.

In conclusion, this study aims to provide new insights into the molecular mechanisms underlying prostate cancer progression through examining the role of oxidative stress and energy metabolism factors. By utilizing scRNA and bulk RNA sequencing, we hope to identify key prognostic factors that can enhance our understanding of prostate cancer and guide the development of personalized treatment strategies.

## RESULTS

### Prostate cancer and oxidative stress and energy metabolism dysfunction

#### Single-cell expression heterogeneity

Single-cell transcriptome analysis can provide deep insights into the cellular functions within tumor tissues, enabling broad applications in studying tumor cell heterogeneity, immunology, bone marrow transplantation, and disease classification.

**Figure 1:**
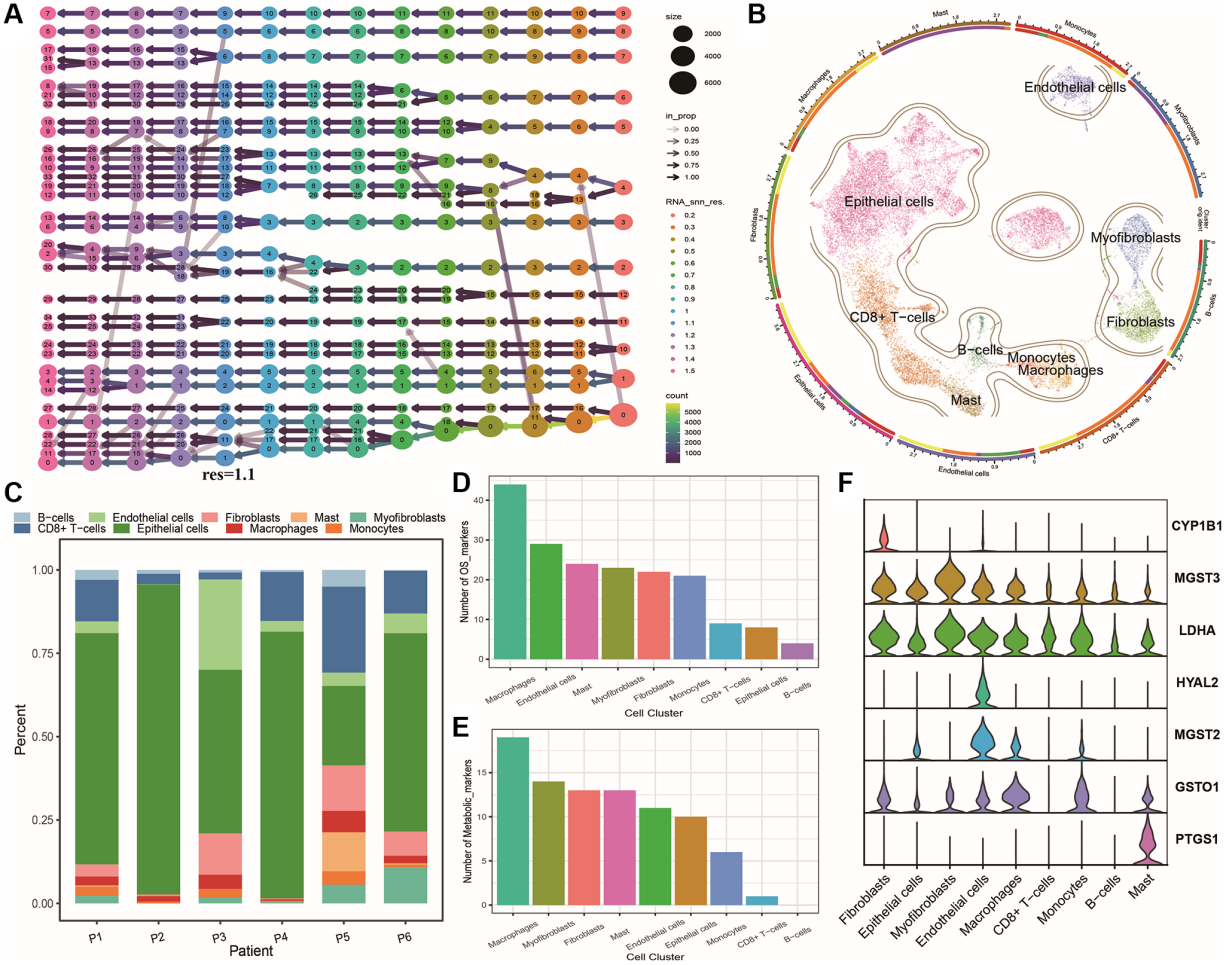
**Heterogeneity of tumor single cells.** (**A**) Cluster dendrogram of single-cell dataset; (**B**) UMAP plots of cell clustering and annotation results for single-cell dataset, with outer circle annotations indicating cell populations and inner circles representing patient origins; (**C**) Cumulative distribution histogram of cell clusters among patients; (**D**) Table showing the number of oxidative stress marker genes in each cell population; (**E**) Table showing the number of energy metabolism marker genes in each cell population; (**F**) Violin plots of OMG marker gene expression in different cell populations.

In this study, we generated a Seurat object by aggregating expression data from six samples and obtained a total of 22,794 cells from prostate cancer tissue after quality control and filtering. After initial data reduction analysis (PCA), we selected the top 10 PCs for further dimensionality reduction and analysis. We then generated clustering results with resolutions ranging from 0.2–1.5, and created a clustering tree as shown in Figure 1A. When the resolution exceeded 1.1, frequent crossovers between clusters appeared. Based on previous analyses and consensus, we used a resolution of 1.1 for cell subtyping. We used SingleR to annotate cell subtypes (Supplementary Table 1: Meta_info) and corrected the annotations using marker genes from databases to obtain nine cell clusters, including B cells, CD8+ T cells, Mast cells, macrophages, monocytes, epithelial cells, endothelial cells, fibroblasts, and smooth muscle cells, as shown in the UMAP plot in Figure 1B. The distribution of these nine cell types across each patient is shown in Figure 1C, which reveals significant differences in the cell type composition among individuals, indicating tumor cell heterogeneity and complexity. We identified oxidative stress markers and energy metabolism markers for each cell cluster by identifying marker genes (Supplementary Table 1: Marker_Table). The number of oxidative stress markers and energy metabolism markers varied among different cell types, with macrophages having the most markers and immune T cells and B cells having the fewest, as shown in Figure 1D, 1E. Finally, we plotted the expression of oxidative stress and energy metabolism genes that intersected among cell types (OMG genes) as a violin plot, which showed strong heterogeneity in their expression across different cell clusters. The expression of OMG genes was highest in endothelial cells, macrophages, and fibroblasts, as shown in Figure 1F.

#### Inter-cellular differences in oxidative stress

**Figure 2:**
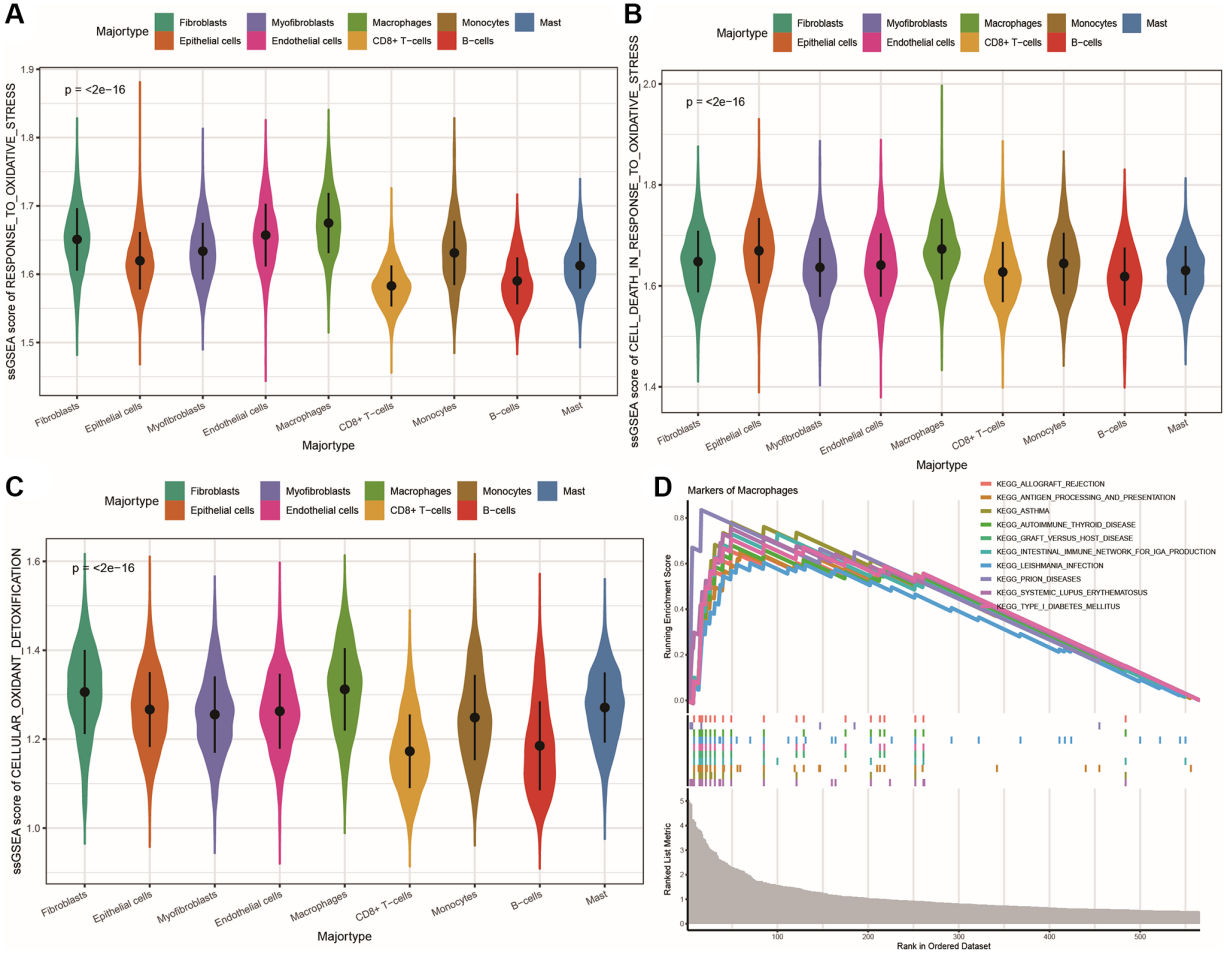
**Differences in oxidative stress and communication between subpopulations of cells.** (**A**–**C**) Violin plots showing the differences in ssGSEA scores for oxidative stress response, oxidative stress-induced cell death, and cellular antioxidant defense among different cell populations; (**D**) Line plot of KEGG pathway GSEA enrichment results for all marker genes of macrophages.

We calculated the ssGSEA (single-sample gene set enrichment analysis) score for each cell’s oxidative stress gene set (Supplementary Table 1: OS_ssGSEA_Score) and compared the differences in oxidative stress function across cell populations using a violin plot. We calculated the ssGSEA score for each cell’s oxidative stress gene set (Supplementary Table 1: OS_ssGSEA_Score) and compared the differences in oxidative stress function between cell populations by plotting a violin plot, as shown in Figure 2A–2C. Macrophages showed strong responses to oxidative stress, oxidative detoxification, and oxidative stress-induced cell death, while CD8+ T cells and B cells had low enrichment scores for all three gene sets. Therefore, we focused on the gene functional features of macrophages. The KEGG GSEA enrichment results of macrophage marker genes (Supplementary Table 1: KEGG_GSEA_Macro) showed that these genes were mainly enriched in pathways related to immunity, such as systemic lupus erythematosus, autoimmune thyroid disease, antigen presentation and processing, and immunoglobulin production, as shown in Figure 2D.

#### Expression characteristics of the oxidative stress active population

**Figure 3:**
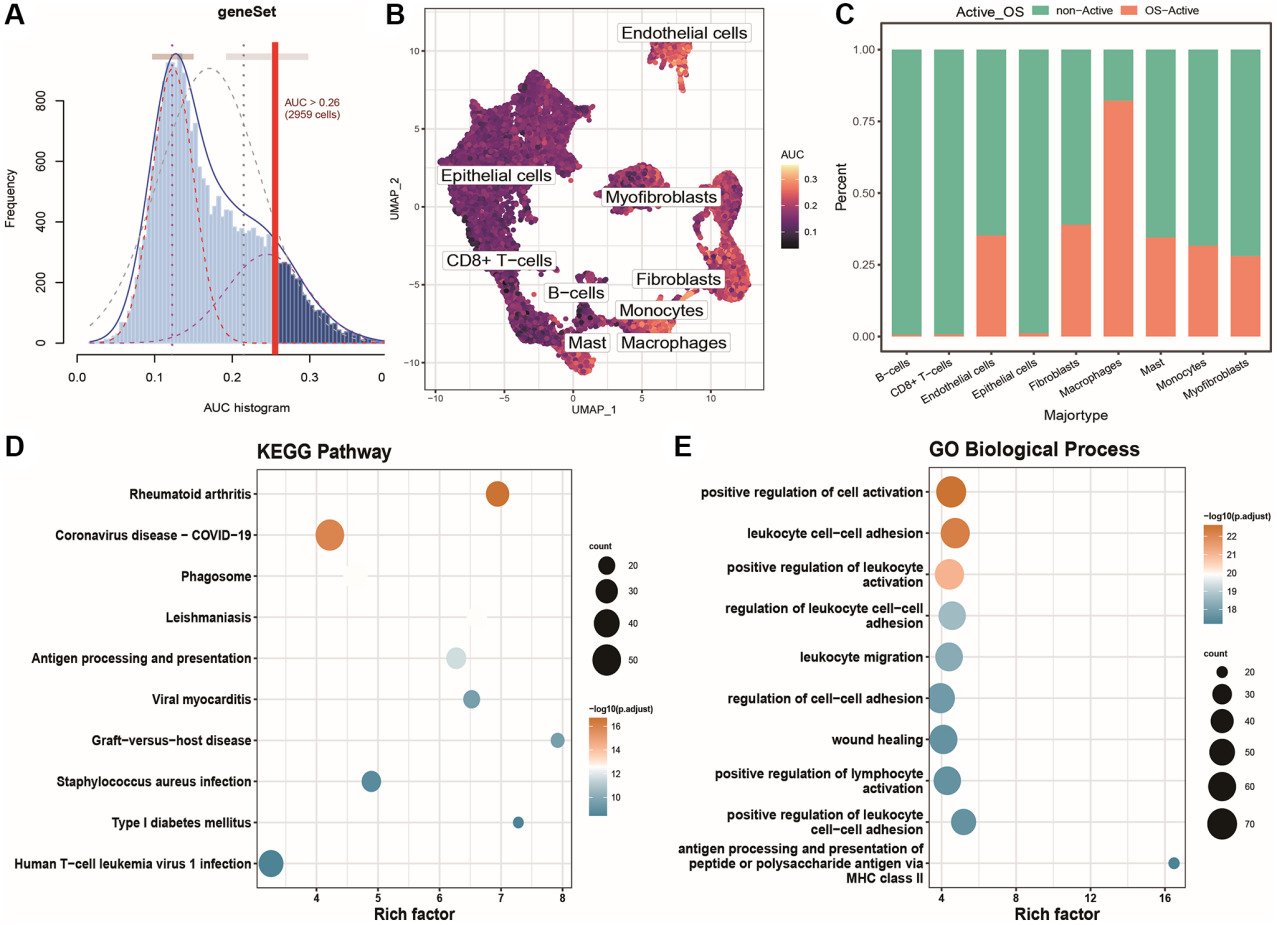
**Identification and functional characterization of oxidative stress-activated populations.** (**A**) AUC scores for specific oxidative stress marker genes; (**B**) UMAP color-coded plot based on cell activity scores, where brighter colors indicate higher response scores and activity levels to the gene set; (**C**) Histogram showing the distribution of active and inactive oxidative stress cell populations in different cell groups; (**D**) Bubble plot of KEGG enrichment results for differentially expressed genes, with bubble size representing the number of enriched genes and color indicating the significance of the enrichment result; (**E**) Bubble plot of GOBP enrichment results for differentially expressed genes.

We obtained 58 intersection genes as oxidative stress-specific markers by taking the intersection of oxidative stress genes and cell subtype-specific marker genes. We then identified the active oxidative stress-related cell population based on the expression of these 58 specific marker genes to study the expression characteristics of oxidative stress-related factors at the single-cell level. The results are shown in Figure 3A: using the optimal threshold (0.26), 2959 cells were identified as the active oxidative stress-related population (Supplementary Table 2: AUC_res). They were mainly distributed among monocytes, macrophages, endothelial cells, and some fibroblasts in the single-cell UMAP plot, as shown in Figure 3B. We then plotted the distribution of active and inactive oxidative stress cells in each cell cluster as a column chart, as shown in Figure 3C. To explore the functional differences between the active and inactive cell populations, we identified 712 differentially expressed genes between the active and inactive oxidative stress populations through differential expression analysis. We then performed KEGG and GO_BP gene set enrichment analyses based on these differentially expressed genes, as shown in Figure 3D, 3E. The enriched pathways identified by KEGG included rheumatoid arthritis, COVID-19, phagosomes, antigen processing and presentation, viral myocarditis, transplant rejection, *Staphylococcus aureus* infection, human T-cell leukemia virus 1 infection, and type 1 diabetes. The enriched functions identified by GO_BP included leukocyte-cell adhesion and regulation, positive regulation of leukocyte activation, leukocyte migration, positive regulation of lymphocyte activation, and biological processes involving MHC class II antigen processing and presentation of peptides or polysaccharide antigens.

#### Expression characteristics of the active energy metabolism population

**Figure 4:**
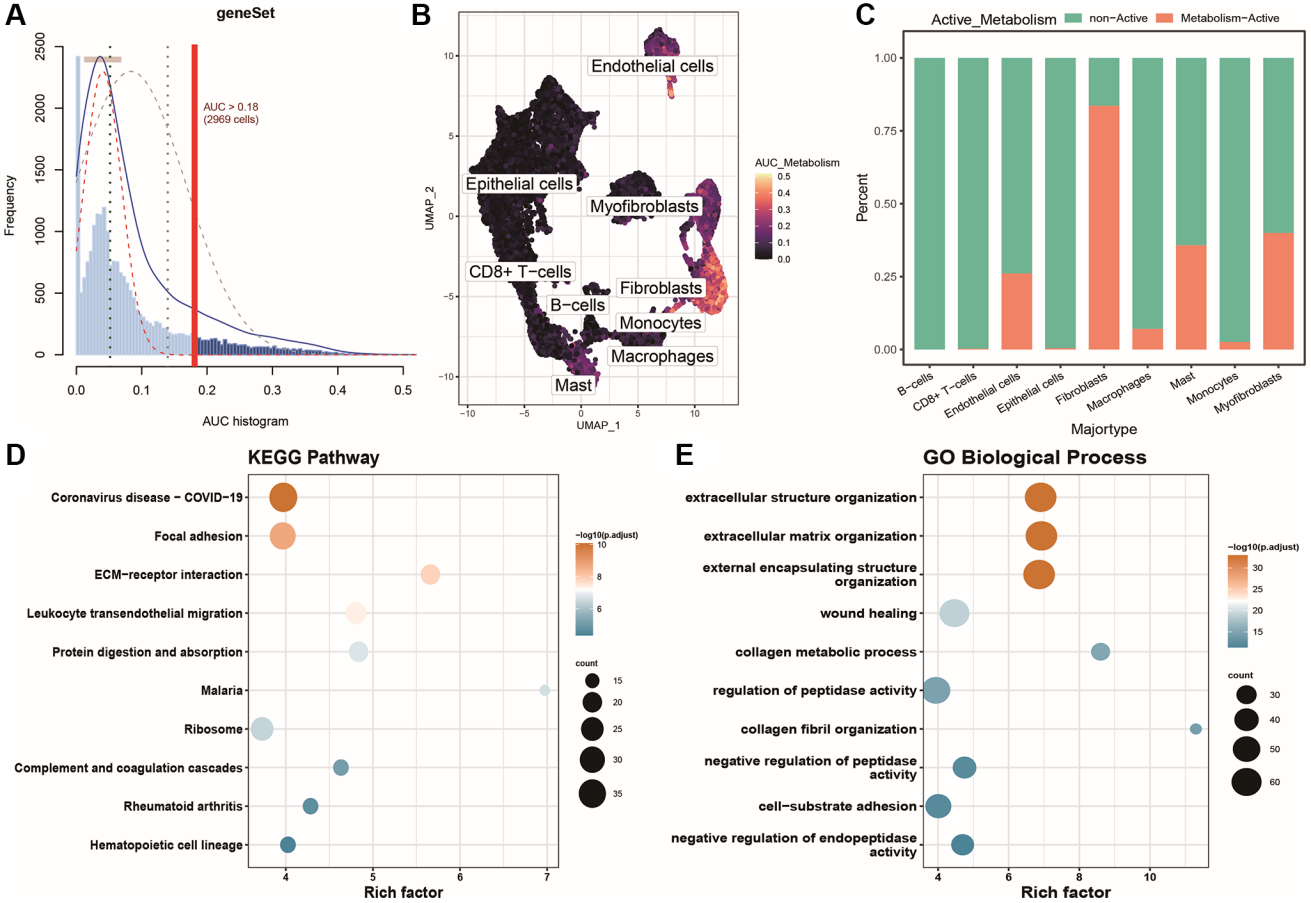
**Identification and characterization of energy metabolism-active populations.** (**A**) AUC scores for specific energy metabolism marker genes; (**B**) UMAP color-coded plot based on cell activity scores, where brighter colors indicate higher response scores and activity levels to the gene set; (**C**) Histogram showing the distribution of active and inactive energy metabolism cell populations in different cell groups; (**D**) Bubble plot of KEGG enrichment results for differentially expressed genes; (**E**) Bubble plot of GOBP enrichment results for differentially expressed genes.

Similarly, we identified the active energy metabolism population based on the expression of 19 specific energy metabolism marker genes. The results are shown in Figure 4A: using the optimal threshold (0.18), 2969 cells were identified as the active energy metabolism population (Supplementary Table 2: AUC_res). They were mainly distributed among fibroblasts and some smooth muscle cells in the single-cell UMAP plot, as shown in Figure 4B. We then plotted the distribution of active and inactive oxidative stress cells in each cell cluster as a column chart, as shown in Figure 4C. Similarly, differential expression analysis identified 656 differentially expressed genes between the active and inactive energy metabolism populations, and KEGG and GO_BP gene set enrichment analyses were performed based on these differentially expressed genes, as shown in Figure 4D, 4E. The enriched pathways identified by KEGG included COVID-19, focal adhesion, ECM-receptor interaction, leukocyte transendothelial migration, protein digestion and absorption, malaria, ribosome, complement and coagulation cascades, and rheumatoid arthritis, as well as the hematopoietic cell lineage. The enriched gene sets identified by GO_BP included cell structure-related processes, wound healing, collagen metabolic processes, regulation of peptidase activity, collagen fibril organization, negative regulation of peptidase activity, cell-matrix adhesion, and negative regulation of endopeptidase activity.

### Tumor expression characteristics and subtype identification in TCGA cohort

#### Differential expression between tumor and normal samples in the TCGA cohort

**Figure 5:**
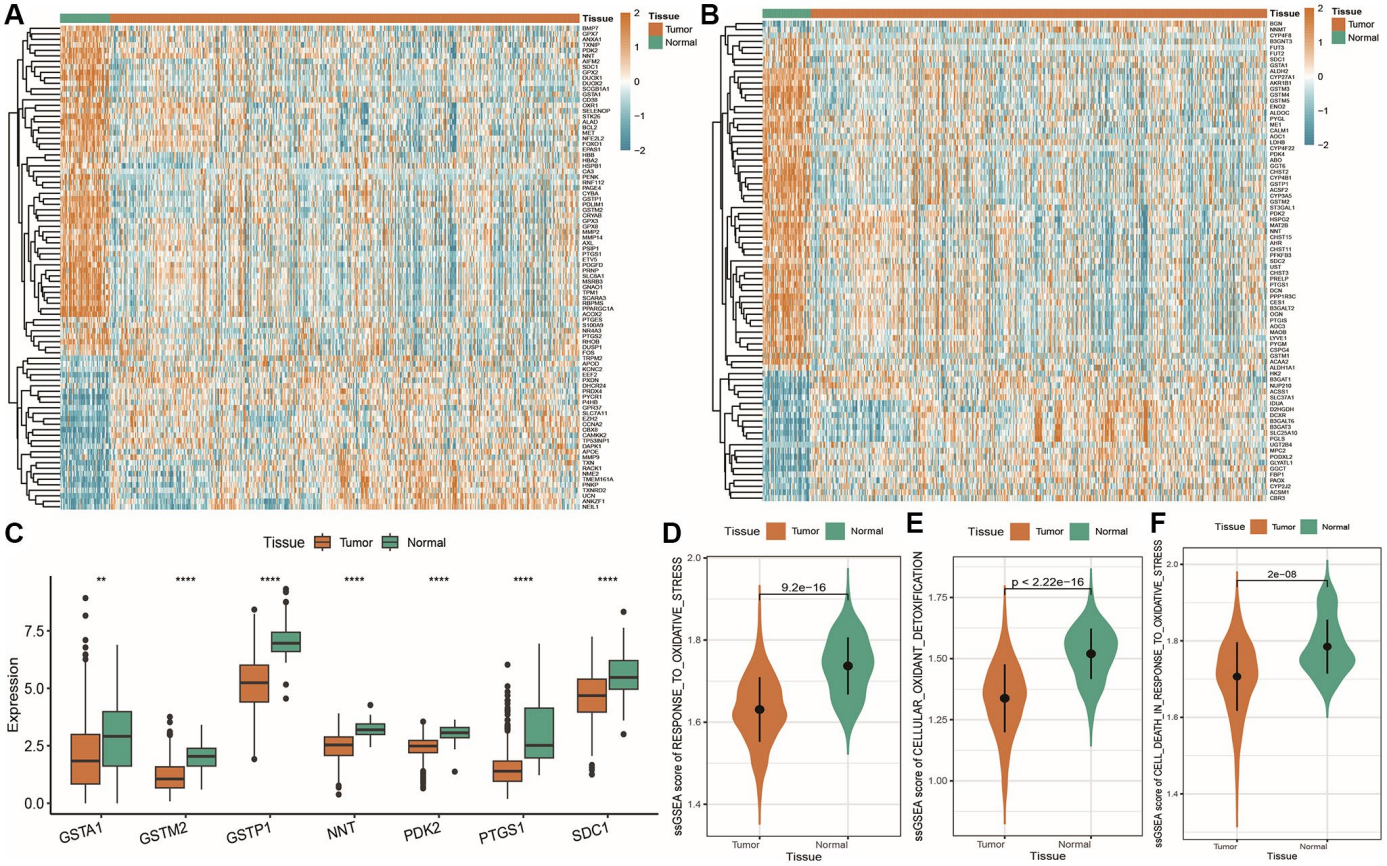
**Dysregulation of oxidative stress and energy metabolism-related genes.** (**A**) Heatmap of significantly differentially expressed oxidative stress genes, with red indicating high expression and blue indicating low expression, and orange and green indicating tumor and normal tissue samples, respectively; (**B**) Heatmap of significantly differentially expressed energy metabolism-related genes; (**C**) Box plot showing the expression of commonly differentially expressed oxidative stress and energy metabolism-related genes, with orange and green representing tumor and normal samples, respectively; (**D**–**F**) Violin plots showing ssGSEA score differences for oxidative stress-related gene sets in tumor and normal tissues, including oxidative stress response, cellular antioxidant defense, and oxidative stress-induced cell death.

To begin, we conducted an analysis of differential gene expression between normal and tumor tissue samples within the TCGA PRAD cohort (Supplementary Table 3: deg_res) and obtained 1974 differentially expressed genes, including 762 upregulated genes and 1212 downregulated genes in tumors. Among all the differentially expressed genes, there were 88 oxidative stress-related genes, and their differential expression heatmap is shown in Figure 5A: among them, 28 oxidative stress-related genes were upregulated in tumors, while 60 were downregulated in tumor tissue. Similarly, among all the differentially expressed genes, there were 81 energy metabolism-related genes, and their differential expression heatmap is shown in Figure 5B: among them, 24 energy metabolism-related genes were upregulated in tumors, while 57 were downregulated in tumor tissue. In addition, there were 7 OMG differentially expressed genes, and their expression patterns are shown in Figure 5C: they were all downregulated in tumor tissue. Finally, we used ssGSEA scores to evaluate the enrichment differences of oxidative stress-related pathways between tumor and normal tissues, as shown in Figure 5D–5F: the oxidative stress response, oxidative detoxification, and oxidative stress-induced cell death in prostate cancer tissue were significantly weaker than those in normal tissue.

#### Identification of potential patients based on differential OM genes

**Figure 6:**
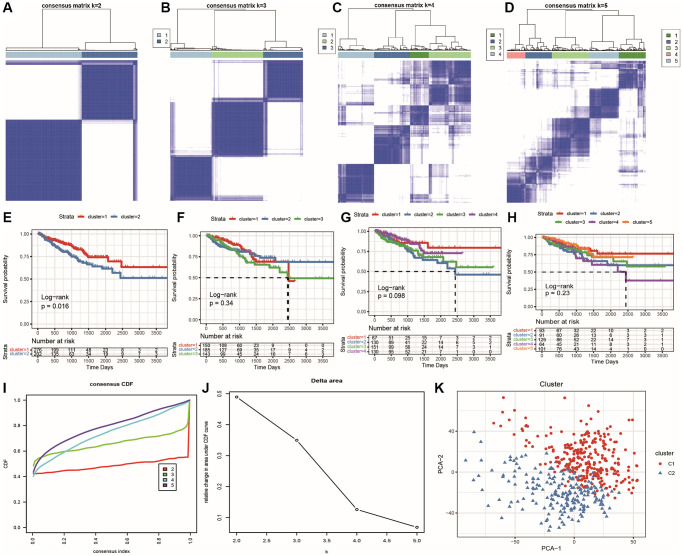
**Identification of prostate cancer subtypes.** (**A**–**D**) Clustering results for k = 2, k = 3, k = 4, and k = 5; (**E**–**H**) Survival curves for k = 2, k = 3, k = 4, and k = 5, with different colors representing different clusters; (**I**) CDF curve distribution for consensus clustering; (**J**) Distribution of area under the CDF curve for consensus clustering; (**K**) PCA scatter plot showing the classification results, with red representing C1 and blue representing C2.

Next, we performed correlation analysis between the differentially expressed oxidative stress-related genes and energy metabolism-related genes, and selected genes that were highly correlated with at least one gene from the other functional group (|correlation|>0.4 and *p* < 0.001). Finally, we obtained a set of 63 genes with differential associations between oxidative stress and energy metabolism, and used them for clustering analysis to identify tumor subtypes. We then plotted Kaplan-Meier survival curves for each cluster subtype. The best clustering result was achieved when using the KM algorithm and Euclidean distance, with a best K value of 2 (Figure 6A–6D), and there was a significant difference in survival curves between the two subtypes (Figure 6E–6H). The sample clustering results are shown in Supplementary Table 4. The consistency cluster cumulative distribution function (CDF) is shown in Figure 6I, which displays the CDF for different values of k. Figure 6J shows the change in the area under the CDF curve ask increases relative to k-1. We then used PCA to reduce the dimensionality of the data and plotted a two-dimensional scatter plot to validate the clustering results, as shown in Figure 6K: the two subtypes were clearly separated, indicating good clustering results. Ultimately, we identified two significant oxidative stress and energy metabolism molecular subtypes with significantly different prognoses, where cluster 1 (C1) had a significantly better prognosis than cluster 2 (C2).

#### Differential expression between tumor subtypes

**Figure 7:**
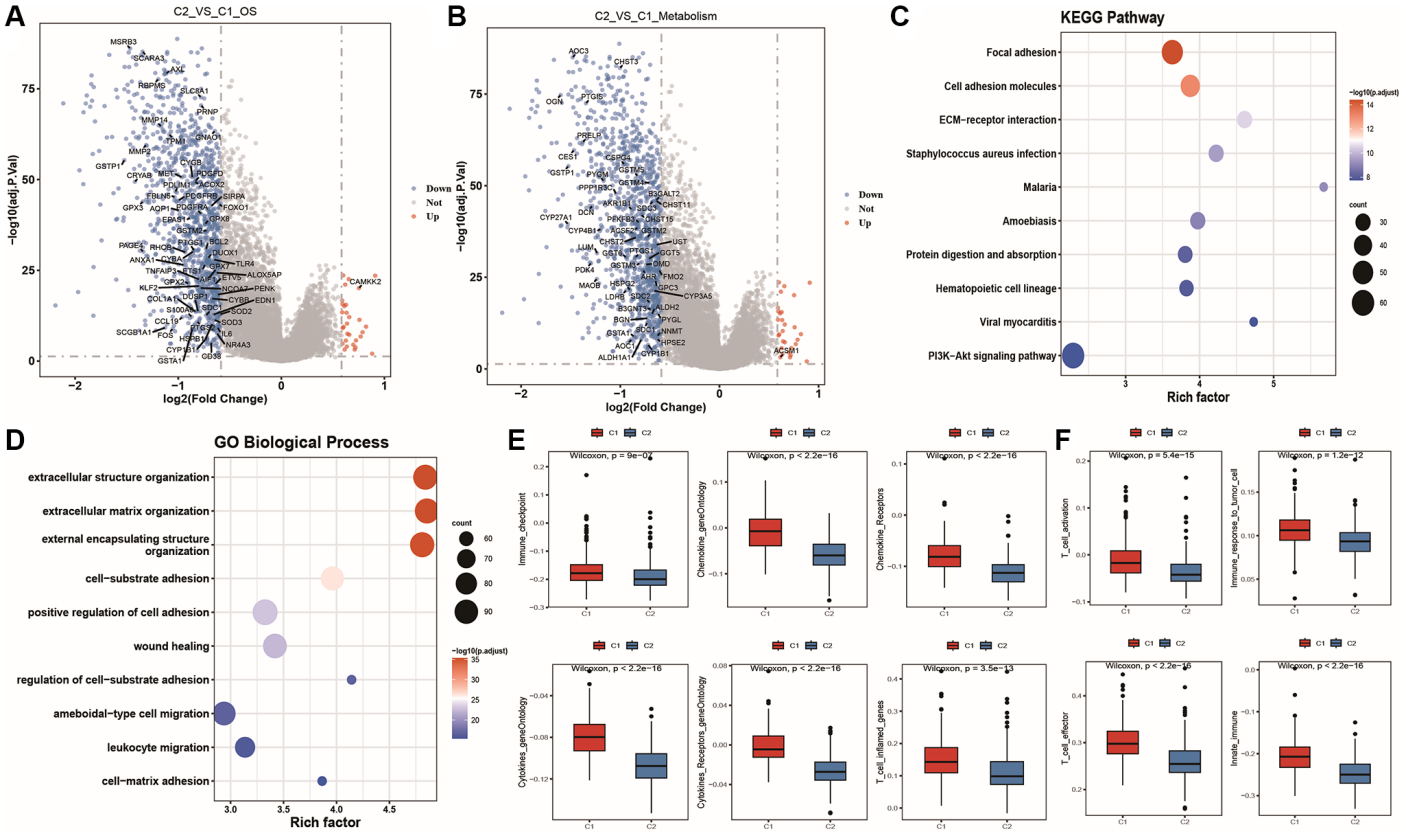
**Differential expression and characterization of molecular subtypes in prostate cancer.** (**A**, **B**) Volcano plots of differentially expressed genes between subtypes, with red indicating upregulation, blue indicating downregulation, and gray indicating no significant difference in gene expression. The labeled genes in the two plots are oxidative stress genes and energy metabolism genes, respectively; (**C**, **D**) Bubble plots of KEGG pathway and GOBP functional enrichment analysis results for differentially expressed genes between subtypes; (**E**, **F**) Box plots showing differences in enrichment scores for immune-related factor gene sets and immune function gene sets between subtypes.

Based on the clustering results of tumor samples, we performed differential expression analysis between the two subtypes and identified 1208 genes with differential expression (Supplementary Table 4: cc_deg). Among them, there were 64 differentially expressed oxidative stress-related genes, and their volcano plot is shown in Figure 7A: only one oxidative stress gene was upregulated in C2, while the rest were upregulated in C1. There were also 52 differentially expressed energy metabolism-related genes, and their volcano plot is shown in Figure 7B: most genes were upregulated in C1. To identify functional differences between prostate cancer molecular subtypes, we performed KEGG and GOBP pathway enrichment analysis based on the differentially expressed genes (Supplementary Table 4) and plotted a bubble chart of the top 10 significantly enriched pathways, as shown in Figure 7C–7D: the differentially expressed genes mainly enriched in functional pathways such as cell adhesion molecules, ECM-receptor interaction, *Staphylococcus aureus* infection, malaria, amoebiasis, protein digestion and absorption, hematopoietic cell lineage, PI3K-Akt signaling pathway, viral myocarditis, and biological processes related to cell structure, cell adhesion regulation, leukocyte migration, and wound healing. We then compared the enrichment differences of six immune-related biological factors using the ssGSEA algorithm, as shown in Figure 7E: C1 had higher enrichment scores than C2. Similarly, we calculated the enrichment of four immune function gene sets, as shown in Figure 7F: C1 had higher immune activity than C2.

#### Differences in immune infiltration between prostate cancer molecular subtypes

The composition of the tumor microenvironment includes stromal and immune cells, which have been proposed as valuable components for diagnosing and prognosticating tumors. In this study, we estimated the proportions of immune infiltrating cells within each tumor sample using four common methods (Supplementary Table 5), and displayed differences in immune infiltration between two subtypes of prostate cancer via box plots. Supplementary Figure 1A depicts the differences in the infiltration proportions of 28 immune cell types calculated by ssGSEA, with 25 cell types exhibiting significant differences in infiltration proportions. Supplementary Figure 1B shows box plots indicating differences in the infiltration proportions of six immune cell types calculated by the TIMER algorithm, where all six immune cell types exhibited significantly higher infiltration proportions in C1 compared to C2. Box plots shown in Supplementary Figure 1C display differences in scores for four metrics calculated using ESTIMATE, with the stromal score, immune score, and ESTIMATE score of C1 being significantly higher than those of C2, while tumor purity was lower in C1 compared to C2. The results for immune infiltrating cell proportions calculated by CIBERSORT and xCell are presented in Supplementary Figures 2 and 3.

### Construction and validation of OMR

#### Identification of prostate cancer prognostic signature

**Figure 8:**
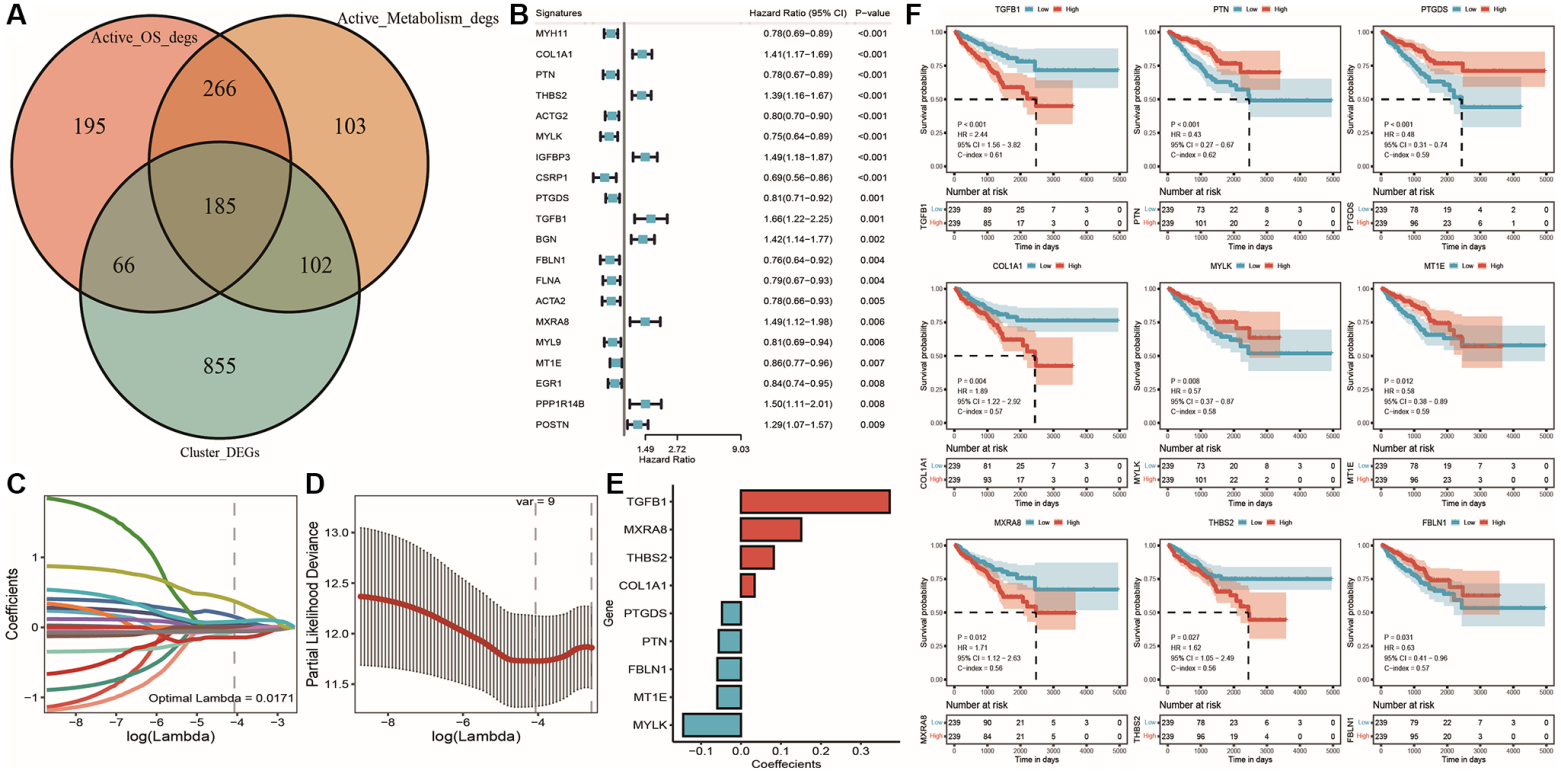
**Lasso regression analysis results for TCGA dataset.** (**A**) Venn diagram of differentially expressed genes between subtypes and active cell populations; (**B**) Forest plot of single-factor Cox analysis results for common differentially expressed genes; (**C**) Trajectory of changes in Lasso regression independent variables, with the x-axis representing the logarithm of the independent variable Lambda and the y-axis representing the coefficient of the independent variable; (**D**) Confidence intervals for each Lambda in Lasso regression; (**E**) Lasso regression coefficients for nine key prognostic factors; (**F**) KM curves for model genes, with red indicating high expression group, blue indicating low expression group, and *p*-value indicating the significance of survival curve differences.

In the process of cancer development, oxidative stress and energy metabolism interact with each other and influence the evolution of tumor cells together. In order to study the impact of both on tumor prognosis, we first identified the intersection of differentially expressed genes identified by three differential analyses (differential expression between TCGA molecular subtypes, differential expression of oxidative stress active cell populations, and differential expression of energy metabolism active cell populations) using a Venn diagram, as shown in Figure 8A: we obtained 185 common DEGs. Then, we analyzed these common differentially expressed genes using univariate Cox analysis and finally identified 20 genes associated with prostate cancer prognosis (Supplementary Table 6: cox_res), as shown in the forest plot of univariate Cox results in Figure 8B. Subsequently, we randomly sampled 2/3 of the TCGA_PRAD overall set (*n* = 478) as the training set (*n* = 320), used Lasso linear regression to remove redundant genes, and set seed = 1110. We ultimately selected 9 prognostic-related signatures (Supplementary Table 6) through this process, as shown in Figure 8C–8E. We utilized the median gene expression value to stratify high and low expression groups, and subsequently constructed Kaplan-Meier survival curves for the selected model genes within the overall TCGA cohort. Remarkably, all model genes exhibited significant differences in KM curves between high and low expression groups as illustrated in Figure 8F.

#### Validation of model performance using TCGA dataset and external dataset

**Figure 9:**
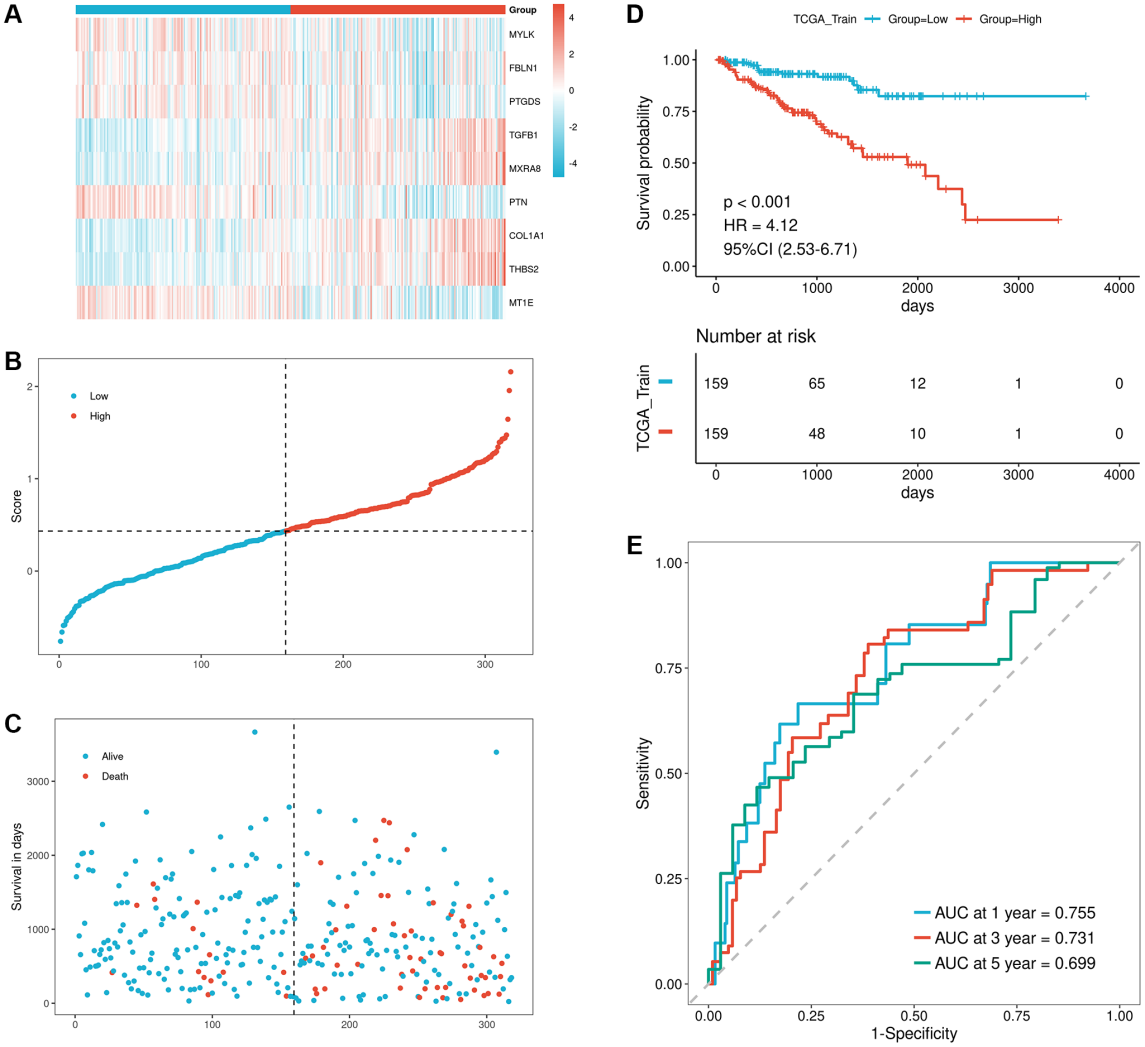
**Validation of prognostic performance of the model using TCGA training set.** (**A**–**C**) Risk triad plots of TCGA training set, including heatmap of model gene expression in risk score groups, scatter plot of risk scores, and scatter plot of survival time, with red representing high-risk group and blue representing low-risk group; (**D**) KM curve of TCGA training set; (**E**) ROC curve of TCGA training set.

To further evaluate the impact of the 9 signatures on the recurrence of the training set, we first calculated the RiskScore for each sample in the dataset using the formula RiskScore = *MYLK* × −0.144 + *FBLN1* × −0.059 + *PTGDS* × −0.047 + *TGFB1* × 0.372 + *MXRA8* × 0.151 + *PTN* × −0.056 + *COL1A1* × 0.034 + *THBS2* × 0.082 + *MT1E* × −0.059 (Supplementary Table 6: TCGA_Train), the median value of RiskScore (0.4329028) was used as a critical threshold to categorize samples into high-risk and low-risk groups. The gene expression heatmap of the model genes is presented in Figure 9A, showing their distribution in the high-risk and low-risk groups of the training set. To observe the relationship between survival and score, scatter plots of survival time, survival status, and sample risk scores were generated (Figure 9B, 9C). Kaplan-Meier curves were also plotted to compare the prognostic differences between the two groups, revealing that the high-risk group had significantly lower survival rates compared to the low-risk group (Figure 9D; *p*-value < 0.001). Moreover, ROC curves were constructed based on the risk model, demonstrating its good predictive capability with AUC values of 0.755/0.731/0.699 for 1/3/5 years (Figure 9E).

To further validate the reliability of the model score in predicting overall survival of prostate cancer patients, we conducted similar analyses on the TCGA PRAD test and overall sets, as well as external datasets GSE70770 and GSE70769, and obtained similar conclusions. These findings suggest the robustness of the model score in predicting overall survival of prostate cancer patients across different datasets (Supplementary Figures 4–7).

### Correlation between risk model and multiple tumor characteristics

#### Independence of risk score

**Figure 10:**
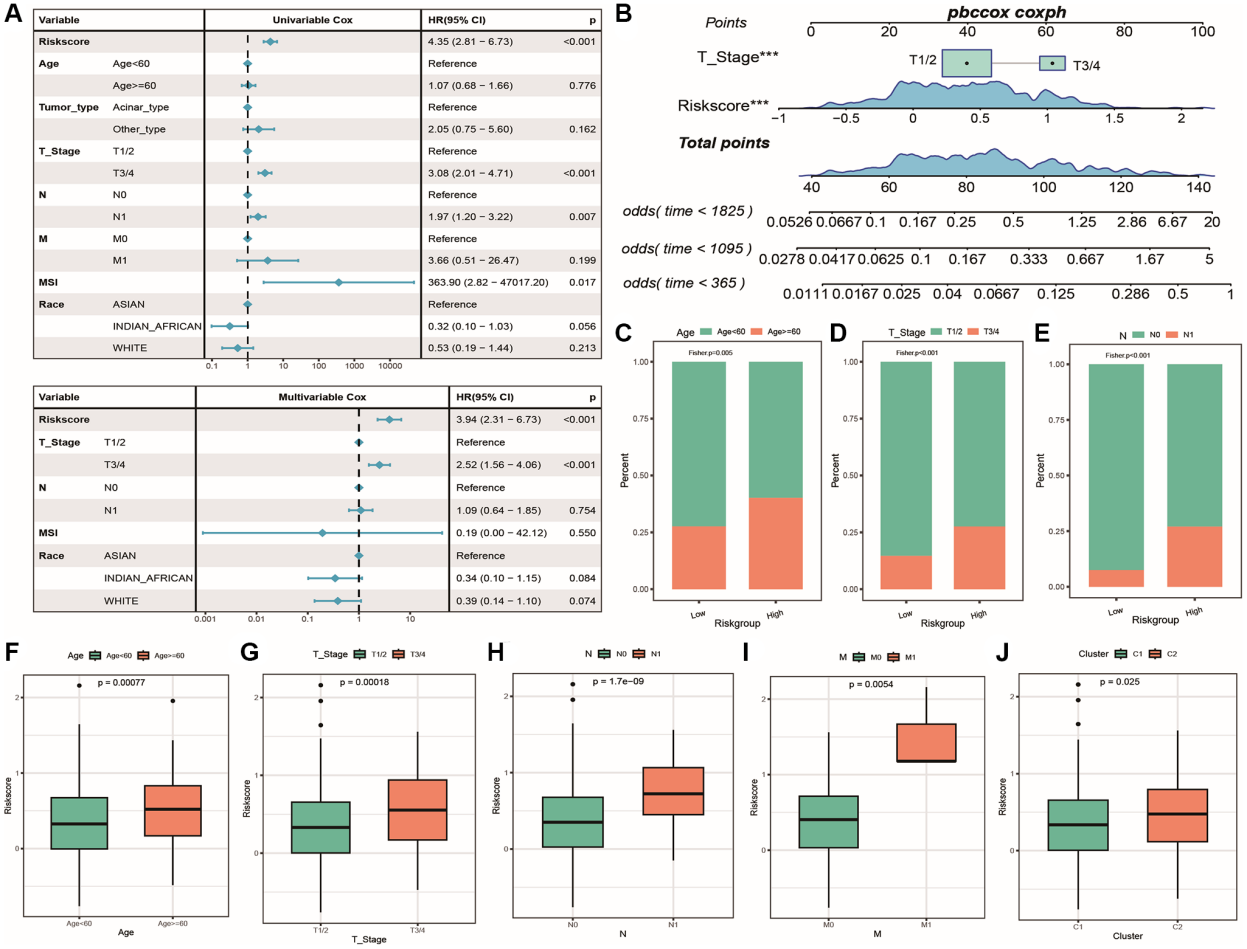
**Independence of risk score with tumor clinical characteristics.** (**A**) Forest plot of single and multiple Cox analysis results for Riskscore and clinical factors in TCGA cohort; (**B**) Column chart of the prediction model, with the square plus line segment representing the contribution of each clinical factor to outcome events, Total Points representing the total score obtained by adding up the scores of all variables, and the three lines at the bottom representing the cumulative survival probability at 5/3/1 years for each value point. (**C**–**E**) Cumulative distribution histogram of different clinical feature groups between high and low-risk groups, with *p*-value indicating the significance of differences; (**F**–**J**) Box plot showing differences in risk score distribution among different clinical feature groups, with different colors representing different groups and *p*-value indicating the significance of differences.

To verify whether RiskScore can serve as an independent prognostic factor, this analysis combined prostate cancer Tumor_type, Age, T_Stage, N (N stage), M (M stage), MSI, and Race information (Supplementary Table 6: Clinical_stat) for single and multiple factor Cox regression analysis. The results are shown in Figure 10A: in the single-factor Cox regression, Riskscore, T_Stage, N, MSI, and Race was significantly different from the Reference. In the multiple-factor Cox analysis, Riskscore and T_Stage was significantly different from the Reference in the presence of other clinical features, proving that they are independent prognostic factors. In addition, a nomogram (Figure 10B) was drawn based on survival time, survival status, Age, and Stage, showing that Riskscore contributed the most as a clinical factor. Then, cumulative distribution plots of Age, T_Stage, and N grouping samples between high-risk and low-risk groups were drawn and Fisher’s exact test was performed (Figure 10C–10E): all *p*-values were less than 0.05, indicating a significant correlation between their distribution and the risk model grouping. We then studied the differences in Riskscore distribution between clinical feature groups, as shown in Figure 10F–10J: Riskscore was significantly different among Age, T_Stage, N stage, M stage, and prostate cancer subtype, indicating that multiple clinical features of cancer are related to the risk score.

#### Differences in immune microenvironment between model groups

**Figure 11:**
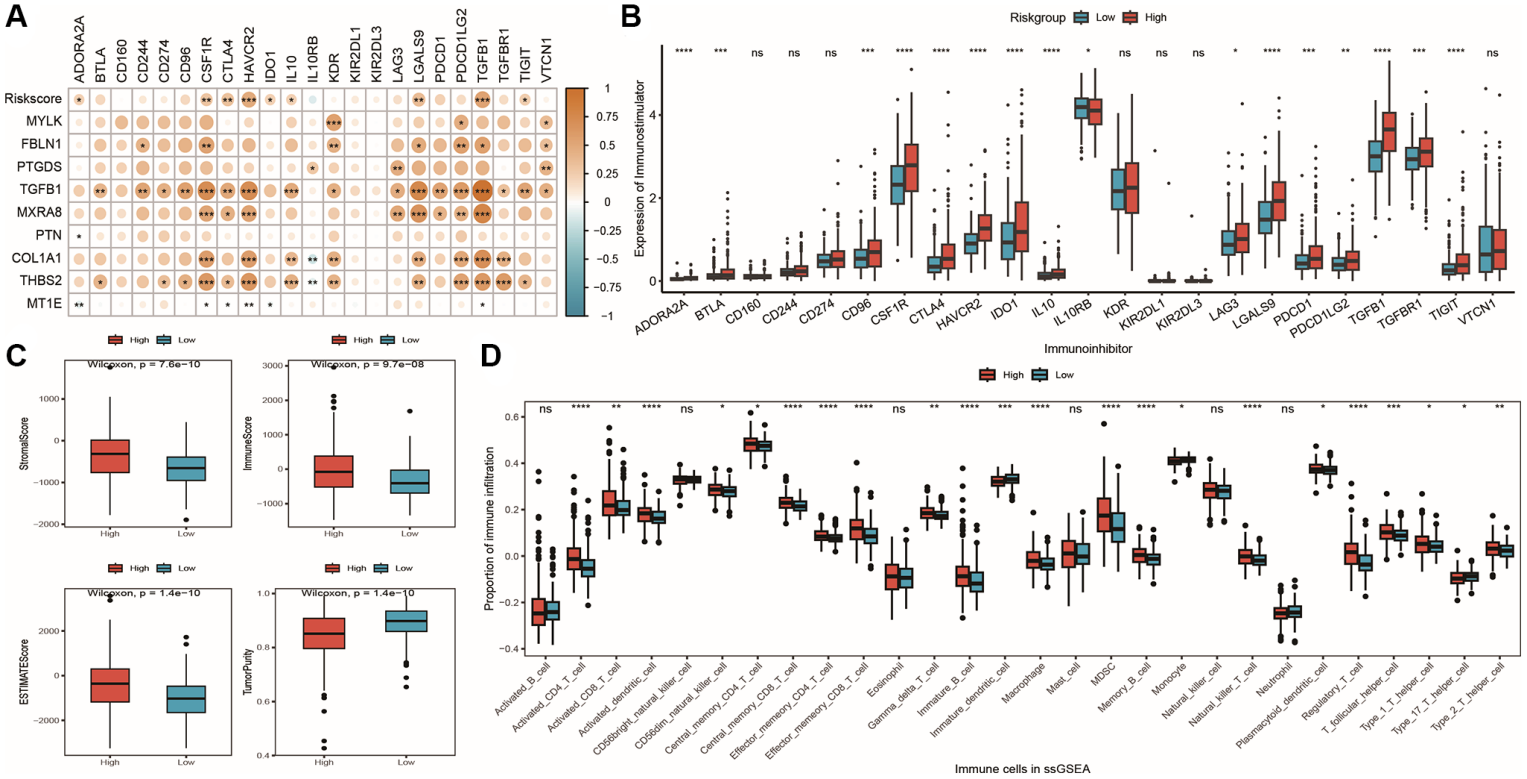
**Differences in immune microenvironment among model groups.** (**A**) Correlation coefficient heatmap between model and immune checkpoint expression, with point color representing the level of correlation and ^*^indicating significance; (**B**) Box plot showing differential expression of 23 immune checkpoint inhibitors between high and low-risk groups; (**C**) Box plot showing differences in stromal score, immune score, ESTIMATE score, and tumor purity, with red and blue representing high and low-risk groups, respectively; (**D**) Box plot showing differences in immune cell infiltration proportions calculated by ssGSEA algorithm between high and low-risk groups, with red and blue representing high and low-risk groups, respectively.

To determine variations in the tumor immune microenvironment between high-risk and low-risk groups, we initially computed associations between Riskscore, the expression of nine model genes, and 23 immune checkpoint inhibitors (Supplementary Table 7). Next, we displayed these correlations as a heatmap (Figure 11A) for visualization purposes. Results demonstrated significant positive correlations between model gene expression and immune checkpoint inhibitor expression for *CSF1R*, *CTLA4*, *HAVCR2*, and *TGFBR1*. We then compared expression differences for 23 immune checkpoint inhibitors between high-risk and low-risk groups, presenting box plots depicting results in Figure 11B: 16 immune checkpoint inhibitors demonstrated differential expression, with higher expression levels observed in the high-risk group. Finally, based on analysis of immune infiltrating cell proportions, we compared differences in immune cell infiltration between high-risk and low-risk groups. Box plots in Figure 11C illustrate a comparison of four ESTIMATE scores between high-risk and low-risk groups. Our results indicated that the high-risk group exhibited higher stromal score, immune score, and ESTIMATE score, but lower tumor purity, compared to the low-risk group, with significant differences observed. Figure 11D depicts differences in the infiltration proportions of 28 immune cell types calculated by ssGSEA between high-risk and low-risk groups, with infiltration proportions of 22 cell types exhibiting significant differences. Additional algorithm results are presented in the supplementary material.

#### Enrichment differences

Based on the HALLMARK pathway enrichment scores of prostate cancer samples (Supplementary Table 8), combined with model grouping information, we explored the pathway enrichment differences between high-risk and low-risk groups, which can help study the relationship between cancer characteristic pathways and prognosis. Supplementary Figure 8A shows that multiple pathways have significantly different enrichment scores between model groups, including fatty acid metabolism, interferon α/γ response, and inflammatory response. We then calculated the correlation between Riskscore and model gene expression and HALLMARK pathway enrichment scores, as shown in Supplementary Figure 8B: RiskScore was significantly negatively correlated with pathways such as fatty acid synthesis, bile acid metabolism, cholesterol homeostasis, and fatty acid metabolism, and significantly positively correlated with pathways such as angiogenesis and G2M checkpoint.

#### Correlation between risk model and prostate cancer genome mutations

**Figure 12:**
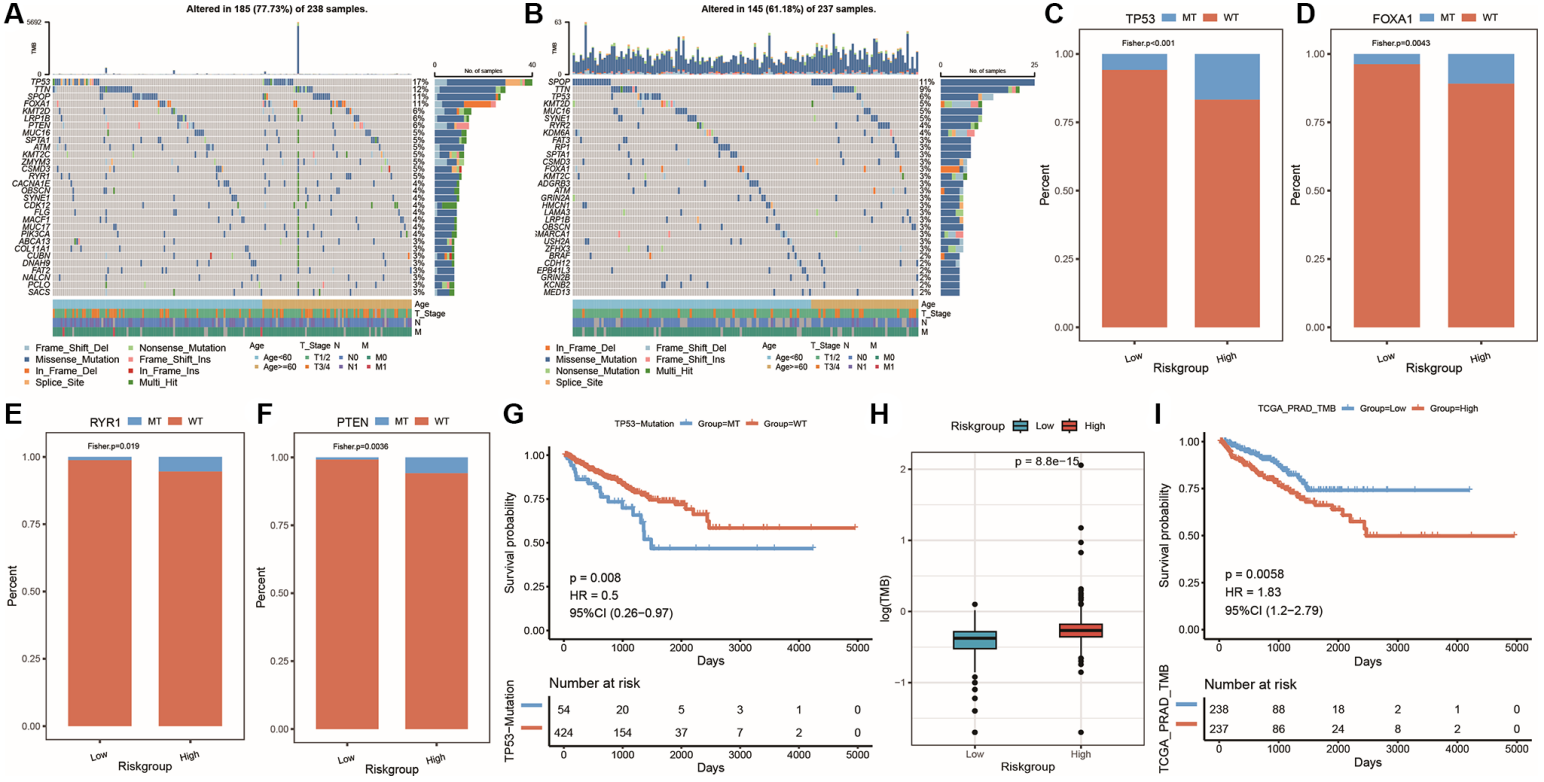
**Genomic mutation differences among model groups.** (**A**) Waterfall plot of the top 30 genes (mutation frequency) with SNV in high-risk group; (**B**) Waterfall plot of the top 30 genes (mutation frequency) with SNV in low-risk group; (**C**–**F**) Cumulative distribution histogram of frequently mutated genes with significant differences between high and low-risk groups; (**G**) Survival curve showing the difference between mutant and wild-type groups of gene TP53; (**H**) Box plot showing the difference in TMB between high and low-risk groups; (**I**) KM survival curve showing the difference between high and low TMB groups, with red representing high TMB group and blue representing low TMB group.

Genomic mutations play an important role in cancer progression and therapy development. To investigate the distribution of somatic variations between high-risk and low-risk groups and gene mutations among samples with different clinical features, we selected the top 30 genes with the highest mutation frequency in both groups and created waterfall plots (Figure 12A, 12B). PRAD featured *TP53*, *TTN*, and *SPOP* as top three genes with the highest mutation frequency, but their mutation frequencies varied between high- and low-risk groups. Comparing mutation frequencies between the two groups revealed significant differences for *TP53*, *FOXA1*, *RYR1*, and *PTEN*, which are genes with high mutation rates, as shown in cumulative distribution bar charts (Figure 12C–12F). TP53 mutation was further associated with prostate cancer recurrence, resulting in worse prognosis (Figure 12G). Finally, we compared tumor mutation burden (TMB) between high- and low-risk groups, revealing higher TMB in the high-risk group (Figure 12H). By dividing samples into high-TMB and low-TMB groups based on median TMB value, we plotted KM survival curves for both groups (Figure 12I), demonstrating significantly worse prognosis and survival differences for patients with high TMB.

### Predicting treatment efficacy using the risk model

#### Evaluating chemotherapy drug resistance using the risk model

**Figure 13:**
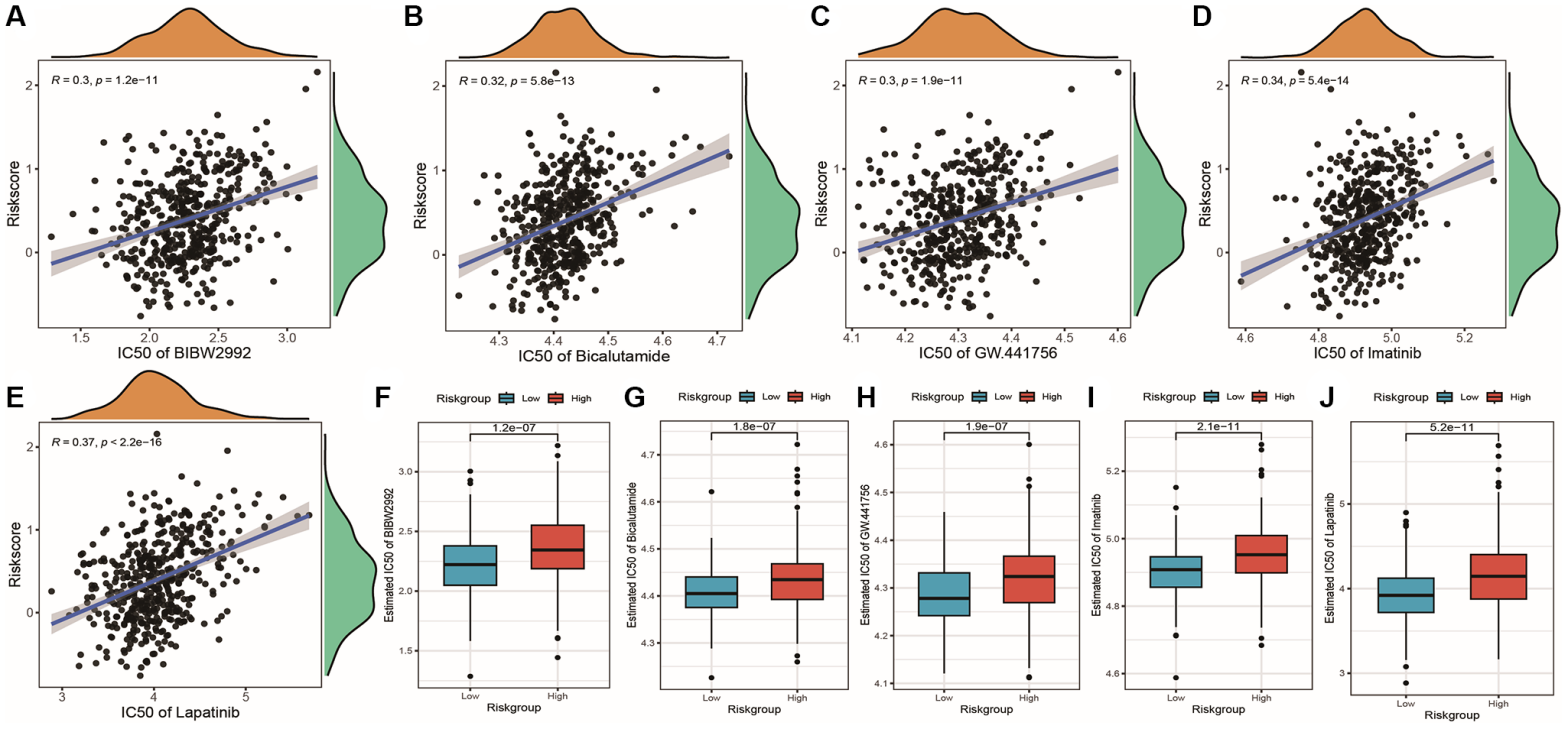
**Correlation between risk score and chemotherapy drug resistance.** (**A**–**E**) Scatter plots showing the correlation between risk score and drug IC_50_ values, with R representing the correlation coefficient and *p*-value indicating the significance of the correlation; (**F**–**J**) Box plots showing differences in drug IC_50_ values between high and low-risk groups.

Using the expression data of TCGA PRAD, we predicted the IC_50_ values of 138 drugs from the GDSC database (Supplementary Table 9). We then performed statistical tests to examine whether there were significant differences in IC_50_ values between high-risk and low-risk groups based on the model grouping results and IC_50_ values. We selected drugs with significantly different IC_50_ values and calculated their correlation with Riskscore, finding that five drugs showed a highly positive correlation with Riskscore (Figure 13A–13E). Next, we plotted box plots displaying the distribution differences of IC_50_ values for these five drugs between high-risk and low-risk groups (Figure 13F–13J). These results indicated that the low-risk group exhibited greater sensitivity to these five chemotherapy drugs, as evidenced by significantly lower IC_50_ values compared to the high-risk group.

#### Predicting the efficacy of immunotherapy using the risk model

**Figure 14:**
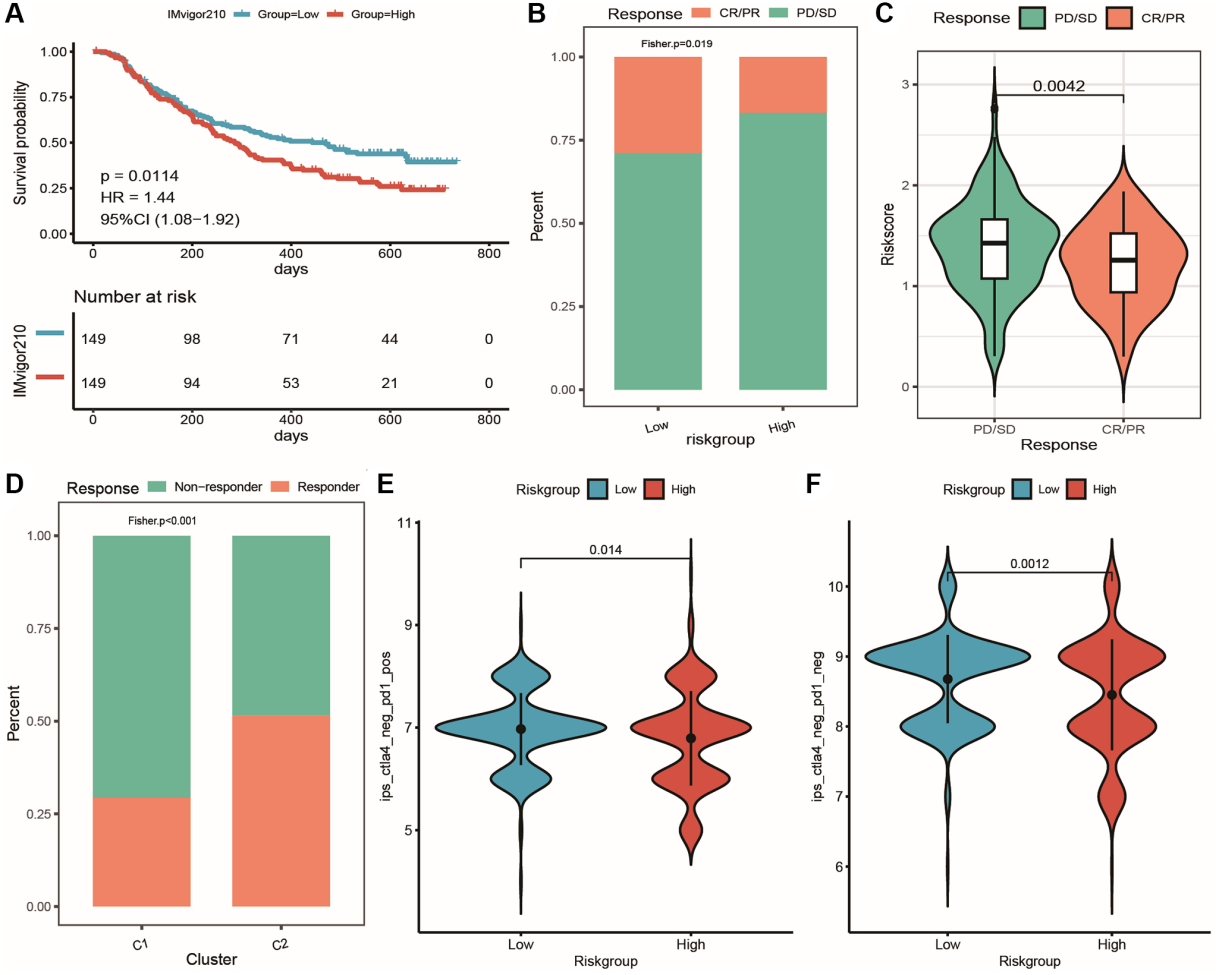
**Prediction of patient response to immunotherapy using the risk model.** (**A**) KM curves of high and low-risk groups in the IMvigor210 cohort; (**B**) Cumulative distribution histogram of response and non-response groups between high and low-risk groups in the IMvigor210 cohort; (**C**) Violin plot showing differences in risk score between response and non-response groups in the IMvigor210 cohort; (**D**) Cumulative distribution histogram of TIDE-predicted immune response between response and non-response groups in the clustering subtype of the IMvigor210 cohort; (**E**, **F**) Violin plots showing differences in IPS score between high and low-risk groups, with red and blue representing high and low-risk groups, respectively.

We started by calculating the risk scores for each sample in the immunotherapy dataset and dividing them into high- and low-risk groups based on median (Supplementary Table 10: IMvigor210_res). Significant survival differences were observed between the two groups using KM curves (Figure 14A). Next, we compared immune response differences between high- and low-risk groups in the immunotherapy cohort, demonstrating that responsive samples were significantly more frequent in the low-risk group than in the high-risk group (Figure 14B). Further analysis revealed a significant difference in risk score between responders and non-responders depicted in Figure 14C, suggesting that the low-risk group is more likely to benefit from immunotherapy. Additionally, we used TIDE online analysis to predict immune response of samples in the TCGA_PRAD dataset and evaluated differences in immune response between oxidative stress and metabolic subtypes (Figure 14D), showing that the proportion of responders was significantly higher in C2 subtype compared to high-risk group and C1 subtype. We also assessed the IPS score to determine the tumor immunogenicity and predict response to immunotherapy. Analysis of four categories of IPS scores between the high-risk and low-risk groups (ips_ctla4_neg_pd1_neg, ips_ctla4_pos_pd1_neg, ips_ctla4_neg_pd1_pos, ips_ctla4_pos_pd1_pos) showed that two IPS scores were significantly higher in the low-risk group, while the other two did not exhibit significant differences between the groups, indicating that patients in the low-risk group are more likely to benefit from immunotherapy (Figure 14E, 14F).

### The role of MXRA8 in prostate cancer cells

**Figure 15:**
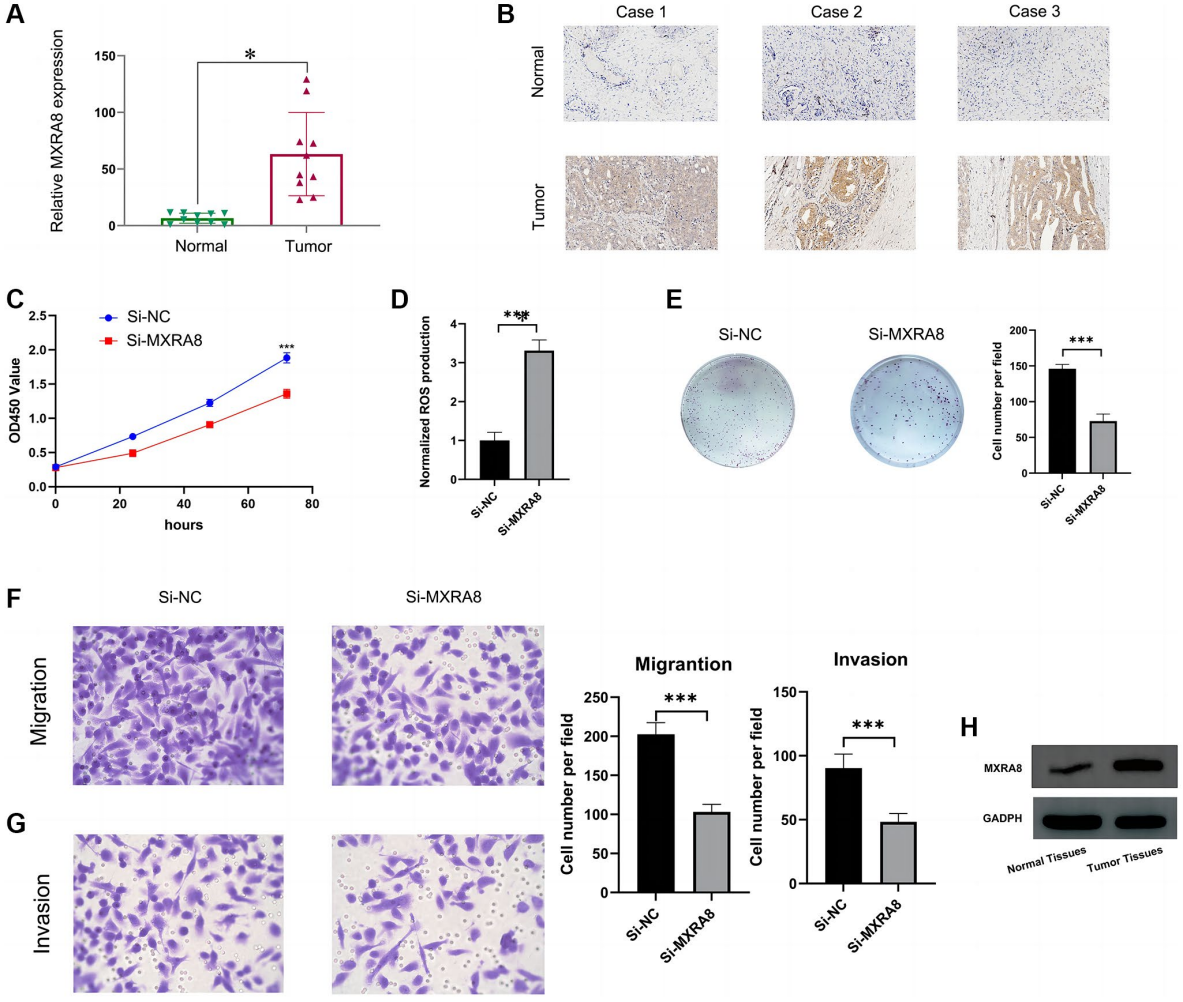
**The role of *MXRA8* in PC-3 cells.** (**A**, **B**) PCR and immunohistochemistry results suggest that *MXRA8* is highly expressed in prostate cancer tissues; (**C**) After knocking out *MXRA8*, the cell viability of PC-3 cells decreased; (**D**) After knocking out *MXRA8*, the ROS production ability of PC-3 cells increased; (**E**) After knocking out *MXRA8*, the proliferation ability of PC-3 cells decreased; (**F**, **G**) After knocking out *MXRA8*, the migration and invasion ability of PC-3 cells decreased; (**H**) The expression of *MXRA8* in pancreatic cancer tissues was higher than that in normal tissues.

**Figure 16:**
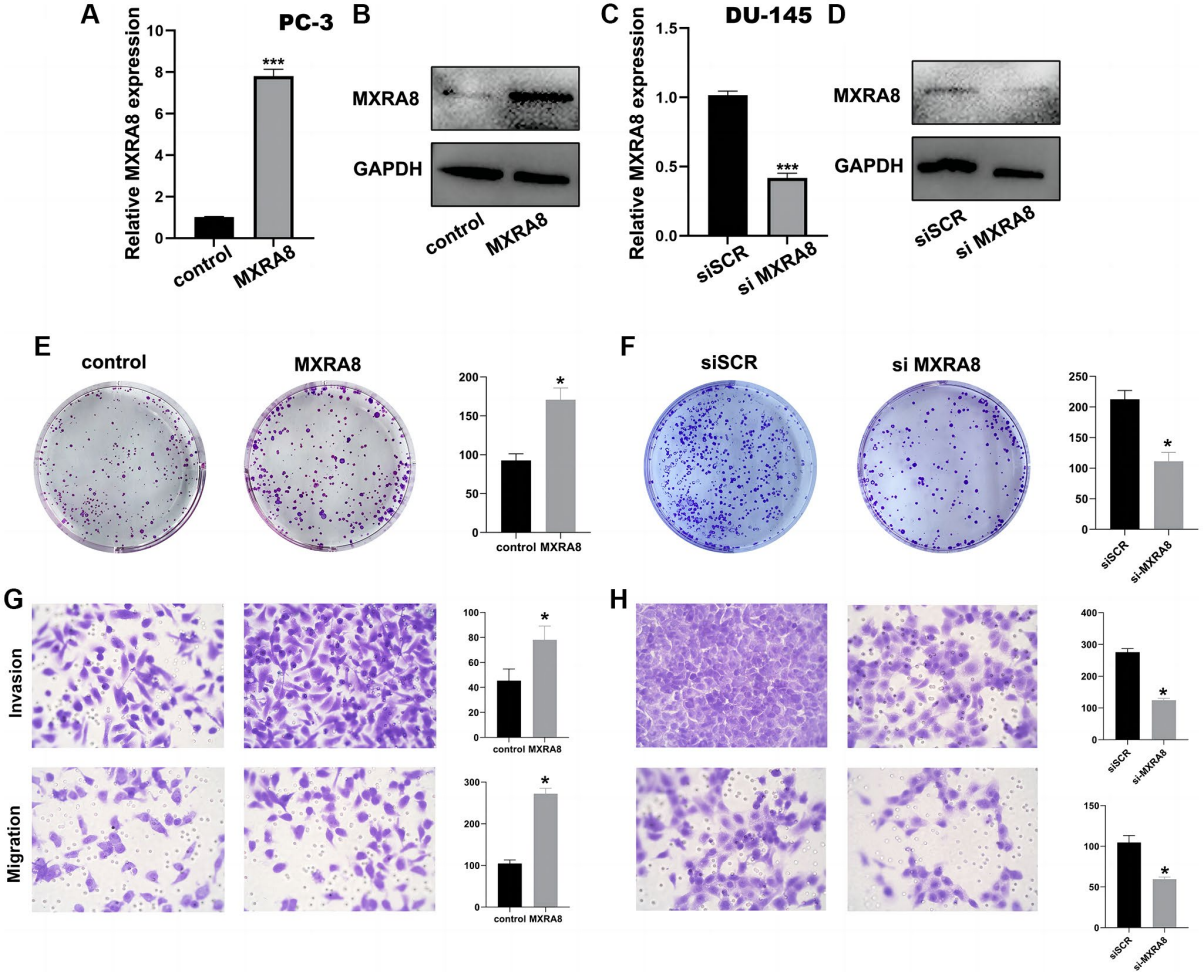
**The role of *MXRA8* in PC-3 cells and DU-145 cells.** (**A**, **B**) After overexpression of *MXRA8*, the mRNA and protein levels of *MXRA8* were up-regulated in PC3 cells; (**C**, **D**) After *MXRA8* knockout, the RNA and protein expression of *MXRA8* in DU-145 cells decreased; (**E**) After overexpression of *MXRA8*, the proliferation ability of PC-3 cells increased; (**F**) After knocking out *MXRA8*, the proliferation ability of DU-145 cells decreased; (**G**) After overexpression of *MXRA8*, the migration and invasion ability of PC-3 cells increased; (**H**) After knocking out *MXRA8*, the migration and invasion ability of DU-145 cells decreased.

This study aimed to investigate the potential role of *MXRA8* in prostate cancer cells. Firstly, we evaluated the expression profile of *MXRA8* in both prostate cancer tissue and adjacent normal tissues using PCR, immunohistochemistry and western blot techniques, where we observed a significant upregulation of *MXRA8* in prostate cancer samples as compared to their normal counterparts (Figure 15A, 15B, 15H). To further unravel the functional significance of *MXRA8*, we employed gene knockout techniques to study its effect on PC-3 and DU-145 cells, the transfection efficiency of siRNA was about 85%. Our results demonstrated that depletion of *MXRA8* had profound effects on the proliferation, invasion, and migration capacities of PC-3 cells, suggesting its critical role in driving cancer progression. Moreover, we observed a reduction in the ROS generation capacity upon *MXRA8* knockdown, indicating its involvement in regulating the redox homeostasis of PC-3 cells (Figure 15C–15G). Then, we overexpressed *MXRA8* in PC-3 cells (Figure 16A, 16B), the proliferation, invasion and migration of PC-3 cells were up-regulated (Figure 16E, 16G). Next, we knock out *MXRA8* in DU-145 cells (Figure 16C, 16D). After *MXRA8* knockout, the proliferation, invasion and migration of DU-145 cells were down regulated (Figure 16F, 16H). Taken together, our findings provide valuable insights into the potential therapeutic targets for prostate cancer treatment by targeting *MXRA8*.

## DISCUSSION

Prostate cancer is a complex disease with significant heterogeneity in terms of clinical presentation, molecular characteristics, and treatment response [13, 14]. In this study, we aimed to investigate the role of oxidative stress and energy metabolism factors in prostate cancer prognosis using scRNA and bulk RNA sequencing. Our results suggest that these factors may play significant roles in tumor progression and patient outcomes.

Our results highlight the importance of intratumoral heterogeneity in prostate cancer. The scRNA sequencing analysis revealed distinct cell subpopulations within the tumors, each with unique gene expression profiles. This suggests that targeting specific cellular subsets may be important for improving treatment efficacy and patient outcomes. Moreover, our machine learning algorithms accurately predicted clinical outcomes in patient samples, demonstrating the potential of scRNA and bulk RNA sequencing in predicting disease prognosis.

Based on bioinformatics analysis and survival data of prostate cancer patients from TCGA and GEO cohorts, a prognostic model was constructed using 9 genes (*MYLK, FBLN1, PTGDS, TGFB1, MXRA8, PTN, COL1A1, THBS2*, and *MT1E*), which was validated by clinical prognosis and correlation with tumor immunity. *MYLK1* gene encodes *MYLK*, a Ca2+/CaM-dependent enzyme that has been identified as a promoter of prostate cancer progression [15–17]. *FBLN* family genes code for ECM proteins responsible for maintaining tissue structure as a component of the basement membrane and elastic fibers [18]. Among them, *FBLN1* is closely associated with tumor cell migration, adhesion, and invasion [19, 20]. *PTGDS* belongs to the lipocalin superfamily of lipid carrier proteins and plays dual roles in prostaglandin metabolism and lipid transport [21]. It is also involved in various cellular processes related to tumorigenesis and progression of solid tumors such as prostate and cervical squamous cell carcinomas [22, 23]. Transforming growth factor β1 (*TGFB1*) is mainly expressed in human tissues and can be synthesized and secreted by almost all cells, often upregulated in tumor cells [24]. Recent studies have shown that *TGFB1* is associated with poor prognosis and risk of progression in metastatic prostate cancer patients but not in non-metastatic disease [25]. *PTN* is a heparin-binding growth factor that performs multiple functions in regulating cell proliferation, migration, and angiogenesis in endothelial cells [26]. Recent studies have found that *PTN* may be a serum biomarker promoting metastatic prostate cancer, and upregulation of *PTN* leads to the migration of prostate cancer cells [27, 28]. Type I collagen α1 (*COL1A1*) is a member of the collagen family that participates in epithelial-mesenchymal transition, which is closely related to malignant tumor development [29]. *COL1A1* expression has been found to promote prostate cancer progression [30]. *THBS2*, which belongs to the matricellular thrombospondin family, interacts with cell receptors, growth factors, and extracellular matrices (ECM) and is involved in regulating processes like cell proliferation, adhesion, and apoptosis [31]. As *THBS2* is upregulated, the survival rate of prostate cancer patients undergoing radiotherapy decreases [32]. Metallothioneins (MTs) are cysteine-rich small proteins that have essential functions in regulating metal homeostasis, protecting against heavy metal toxicity, preventing oxidative stress and DNA damage, as well as contributing to tumor development, drug resistance, and progression [33]. Studies have shown that decreased expression of *MT1E* promotes the progression of prostate cancer [34]. In addition, no association has been found between *MXRA8* and prostate cancer prognosis or its role as a novel marker for prostate cancer. Next, we investigated the function of *MXRA8* gene in prostate cancer. Our findings indicate that *MXRA8* is overexpressed in prostate cancer tissue and its knockout leads to a significant reduction in proliferation, invasion, migration, and ROS generation capacity of PC-3 cells. These results suggest that *MXRA8* plays a crucial role as an oncogene in prostate cancer.

Identifying key prognostic factors associated with aggressive phenotypes and poor prognosis is critical for developing personalized treatment strategies. Our findings may aid in the development of new therapeutic targets, such as targeting glycolysis or oxidative stress-related pathways. Additionally, the identification of specific molecular signatures associated with poor prognosis may inform clinical decision-making and guide treatment choices.

One limitation of our study is the relatively small sample size. Further validation in larger patient cohorts is necessary to confirm our findings and identify additional prognostic factors. Additionally, the changes in gene expression observed in our study may not necessarily translate into functional changes at the protein level. Therefore, further investigation is needed to elucidate the mechanistic links between altered oxidative stress and energy metabolism pathways and prostate cancer progression.

In conclusion, our study provides new insights into the role of oxidative stress and energy metabolism factors in prostate cancer prognosis. Utilizing scRNA and bulk RNA sequencing allowed us to identify specific pathways associated with aggressive phenotypes and poor prognosis, as well as intratumoral heterogeneity that may influence treatment efficacy. Our findings have important implications for developing personalized treatment strategies and improving patient outcomes. Further research is needed to validate our results in larger patient cohorts and investigate the clinical applications of scRNA and bulk RNA sequencing in prostate cancer diagnosis and treatment.

## CONCLUSIONS

**Figure 17:**
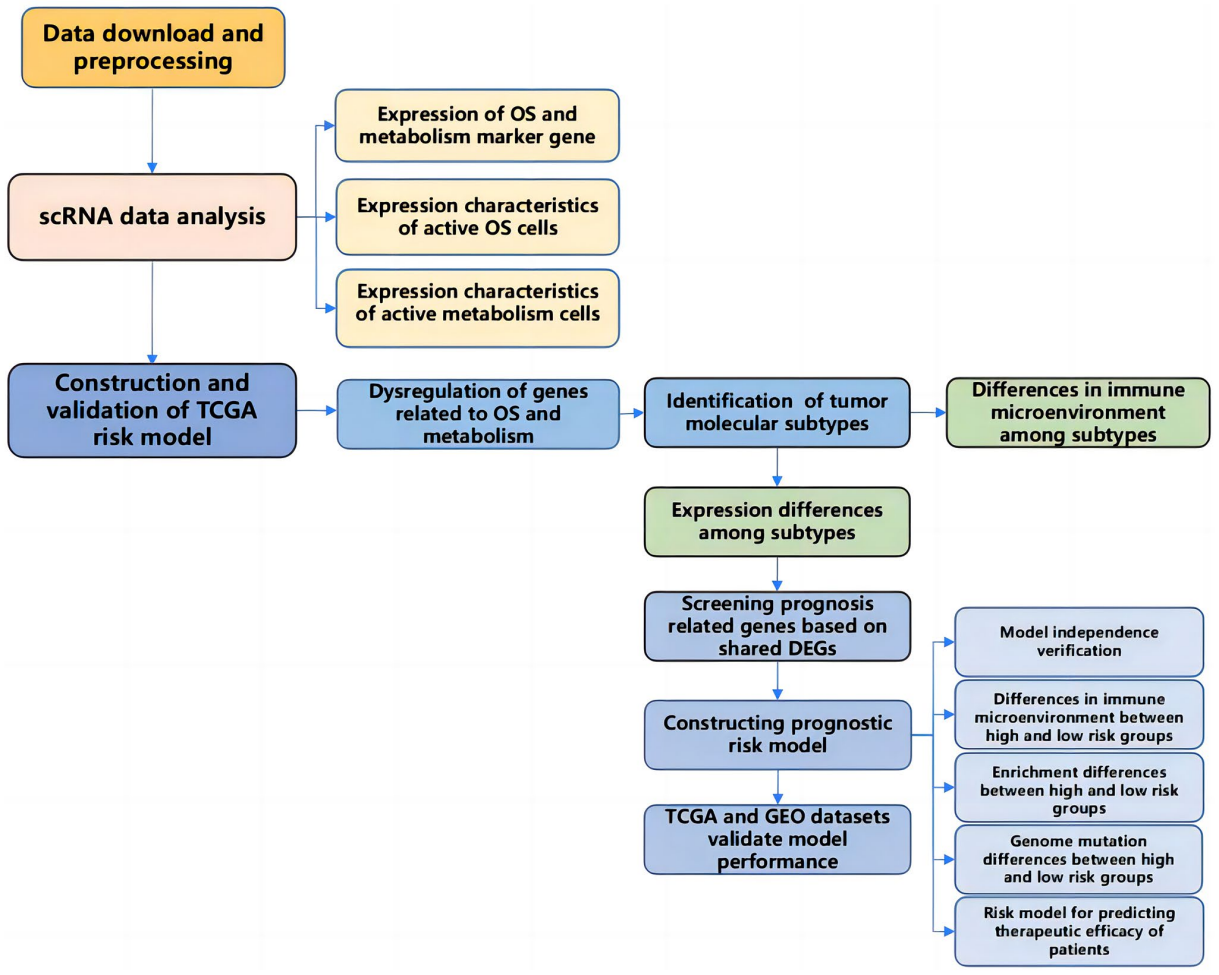
The workflow diagram of this present study.

In this study, we characterized the heterogeneous expression of oxidative stress and energy metabolism-related genes in a single-cell dataset and identified two functionally active cell populations and their corresponding differentially expressed genes. Based on the dysregulated oxidative stress and energy metabolism-related genes in tumors from TCGA PRAD, we identified two molecular subtypes of prostate cancer and analyzed their survival, immune function, and gene expression. Then, we constructed a risk score model (OMR) to evaluate the prognosis of prostate cancer based on the selection of common differentially expressed genes. The model was validated using internal training and testing datasets, an overall dataset, and two external validation datasets, demonstrating good and stable prognostic evaluation performance. We also investigated the relationship between the risk score model and the tumor immune microenvironment, genomic variations, chemotherapy resistance, and immune response, and found that model genes may serve as markers for prostate cancer immune response (Figure 17).

## METHODS

### Expression data download and preprocessing

The TCGA PRAD dataset was utilized for survival analysis, with FPKM expression profiles transformed to log2(FPKM+1) and mutation data analyzed for genomic mutations (Supplementary Table 11). For analysis of single-cell datasets, the Seurat R package was used to construct objects from expression matrix and sample information files downloaded from GEO (accession number GSE137829). External validation sets for risk models were obtained from GEO datasets GSE70770 and GSE70769. The IMvigor210 dataset was used as an immune therapy dataset; its expression and clinical information were downloaded from http://research-pub.gene.com/IMvigor210CoreBiologies, and raw count data were standardized using DEseq2 before being converted to TPM values.

### Gene set download

Three oxidative stress-related GO pathways were collected from the Molecular Signature Database v7.40 (MsigDB), and 481 oxidative stress-related genes were obtained from three gene sets. The energy metabolism gene set was collected from reference PMC9262104, and contains 594 genes from 11 Reactome pathways (Supplementary Table 12).

### Single-cell data analysis

Seurat R package was used for single-cell transcriptome analysis. Single-cell sequencing results of primary tumors and liver metastases were integrated into a Seurat object. Cells and genes were filtered based on count, doublet_cutoff, mitochondrial gene proportion, and red blood cell reads proportion. High variable genes were selected for subsequent analysis. Batch effects were removed using RunHarmony(). Cell subtypes were identified with FindClusters() and annotated using SingleR with BlueprintEncodeData as the reference database. Marker genes were identified using FindAllMarkers(). Data visualization was performed using DimPlot, DotPlot, and VlnPlot.

### Differential expression analysis

The R package limma was used to perform differential expression analysis of samples from different tissue sources and clustering subtypes of TCGA PRAD cohort, with a threshold of adj.*p*-value < 0.05 and |log2FC| > 0.585 (1.5 times the FoldChange value as cutoff) after Benjamini-Hochberg (FDR) correction for selecting differentially expressed genes.

### Unsupervised clustering analysis

Prostate cancer subtypes were identified using the ConsensusClusterPlus R package, which employed a resampling rate of 80% and 1000 repetitions to ensure clustering stability. Consensus clustering analysis was conducted on PRAD samples, and KM survival curves were generated between subtypes with the survival R package. The significance of survival differences was determined using a Log-rank test. The final subtype classification was based on a clustering result that exhibited good performance and significant survival differences between subtypes.

### Construction of OMR

In this study, common differentially expressed genes (common_degs) were identified between different clusters, active and inactive oxidative stress cell populations, and active and inactive immune cell populations. Single-factor Cox analysis was performed on these intersection genes to screen for tumor prognosis-related genes using a *p*-value threshold of < 0.05. A prognostic risk score model for prostate cancer was then constructed based on the cox-selected genes. For variable selection in the risk model, the Lasso (Least absolute shrinkage and selection operator) method developed by Tibshirani (1996) was employed to decrease the number of genes. Finally, a multivariable Cox regression model was employed to construct the prostate cancer risk score model, with the calculation formula presented below:


$$\text{Riskscore}\;\text{=}\;\sum_{i\;=\;0}^{n}\text{βi}\;\text{×}\;\text{χi}$$

βi: The weight coefficient of each gene; χi: The expression level of each gene.

To evaluate the predictive ability of the perturbation scoring model, ROC (receiver operating characteristic) curves were generated using the timeROC R package. The ggplot2 R package was employed to create scatter plots of survival time and status, in addition to sample scores. The tumor samples were categorized into high- and low-risk groups using the median risk score threshold, and prognostic survival curves were constructed using the Kaplan-Meier method. The log-rank test was employed to evaluate any significant differences in survival between these two groups.

### KM curve for gene expression

The Kaplan-Meier method was employed to generate prognostic survival curves based on gene expression data obtained from PRAD tumor samples. The samples were divided into high- and low-expression groups using the median expression level as the threshold, and differences in survival between these two groups were assessed via log-rank test. Genes with a *p*-value less than 0.05 were considered significantly associated with PRAD prognosis.

### Evaluation of immune cell infiltration ratio

Immune cell infiltration ratios were calculated based on the TCGA PRAD dataset using multiple methods, including CIBERSORT, ESTIMATE, TIMER, xCell, and ssGSEA. CIBERSORT employed a leukocyte feature gene matrix LM22 to differentiate 22 types of immune cells. ESTIMATE provided immune score, tumor purity, stromal score, and ESTIMATE score calculations. TIMER estimated the abundance of six immune cell types via deconvolution methods. xCell performed cell-type enrichment analysis utilizing gene expression data of 64 immune cells and stromal cell types, with machine learning applied to reduce correlation between closely related cell types.

### Gene set enrichment analysis

The ssGSEA algorithm of the R package GSVA was used to calculate the enrichment scores for HALLMARK pathways and immune-related gene sets based on the gene expression data of prostate cancer samples from TCGA. The differences in enrichment scores between groups were calculated using box plots and statistical tests. To examine the relationship between the Risk score and the enrichment score, we utilized the cor() function to calculate the correlation and visualized the results using the R package corrplot.

### Genomic SNV analysis

Utilized the maf file of somatic mutation detection results from the TCGA PRAD cohort to generate a waterfall plot via the oncoplot() function of the R package maftools, which demonstrated differences in SNV mutations across different groups. Differential statistics on mutated genes between high- and low-risk groups were performed using the mafCompare() function, with a cumulative distribution bar graph used to visualize differences in mutations between these two groups. Tumor mutational burden (TMB) was also calculated for each sample to investigate the relationship between model grouping and TMB.

### Drug sensitivity

Utilized gene expression data from the PRAD cohort combined with the GDSC database to predict sensitivity (IC_50_ value) of 138 drugs using the pRRophetic R package. Differences in IC_50_ values between high- and low-risk groups were examined via box plots and statistical tests. Significant drugs were selected for heatmap generation to explore the relationship between the model and chemotherapy drugs. The Spearman algorithm was employed to calculate correlation between drug IC_50_ value and gene expression in the model or Risk score, with scatter plots utilized to display the correlation between the IC_50_ value and Risk score for drugs exhibiting strong correlation.

### Prediction of immune therapy response

The Tumor Immune Dysfunction and Exclusion (TIDE) score, available at http://tide.dfci.harvard.edu, is a computational algorithm that predicts immunotherapy response based on pre-treatment tumor expression profile. This algorithm simulates various tumor immune escape scenarios with different levels of cytotoxic T-cell infiltration by scoring multiple published transcriptomic biomarkers. The TIDE score combines both T-cell dysfunction and exclusion features, leading to superior predictive ability relative to other biomarkers. Additionally, the TIDE score remains stable across varying levels of tumor-infiltrating T-cell cytotoxicity. Alternatively, the Immunophenoscore (IPS) score is used to evaluate tumor immunogenicity and predict its response to immunotherapy in various cancer types. IPS scores for PRAD tumor samples were obtained from the TCGA dataset via an online website (https://tcia.at/home), with statistical tests employed to compare scores between groups.

### Cell culture and transfection

The PC-3 cell line and DU-145 cell line (Prostate cancer cell line) was acquired from the Chinese Academy of Sciences and cultured in DMEM high-sugar medium comprising 10% fetal bovine serum (FBS; GIBCO) and 1% penicillin-streptomycin. Cells were cultured in a humidified incubator under 5% CO_2_ at 37°C. One day prior to transfection, PC-3 and DU-145 cells were cultured in six-well plates to achieve 50–60% confluence. For *MXRA8* overexpression, the sequence was cloned into the pcDNA3.1 vector and packaged using pMD2.G and psPAX2. Transfection of the MXRA8-targeted pEZ-M03 vector was carried out using Lipofectamine 2000 as per the provided guidelines [35].

### Measurement of intracellular ROS levels

Intracellular ROS levels were assessed using a Reactive Oxygen Species Assay Kit (Beyotime Biotechnology, China), which employed 2′,7′-dichlorofluorescein-diacetate (DCFH-DA) as the primary component. DCFH-DA is rapidly oxidized by intracellular ROS to produce fluorescent dichlorofluorescein (DCF). To measure DCF levels, cells were exposed to various DET concentrations and time intervals, then seeded into 96-well plates and incubated with DCFH-DA at 37°C for 20 minutes, following established protocols.

### RNA isolation and RT-PCR analysis

Total RNA was extracted using RNAiso Plus (9108, Takara, Japan). The mRNA was reversely transcribed into complementary DNA with PrimeScript RT Master Mix (RR036A, Takara, Japan). The expression levels of *MXRA8* mRNA were detected via TB Green^®^ Premix Ex Taq™ II (RR820Q, Takara, Japan). GAPDH or U6 were applied as internal controls. The relative expression levels of genes were calculated with the 2^−ΔΔCt^ method. The primers used for upstream and downstream sequences of *MXRA8* were 5′-TAACTTGGCGGAGTTCGCTGTG-3′ and 5′-CTAGGTCGATGTACTTGGCAGG-3′, respectively.

### Patients and tissues

All of the experiments were approved by the Institutional Review Board and Ethics Committee of the First Hospital of China Medical University. And all patients signed an informed consent form. Prostate cancer tissues and corresponding adjacent tissues were collected and stored at −80°C. None of the patients had received preoperative chemotherapy or radiotherapy. We collected 10 pairs of prostate cancer and corresponding adjacent tissues.

### IHC (immunohistochemistry) analysis

Tissue fixed in 4% paraformaldehyde, then embedded in paraffin. Tissue specimens were successively incubated with antibodies against Ki-67, a biotin-conjugated secondary antibody and an avidin-biotin-peroxidase complex. Visualization was performed using amino-ethyl carbazole chromogen. Slides were analyzed using the Olympus BX43 microscope system (Olympus, Japan).

### CCK8 assay

CCK-8 assay was used to measure the cell proliferation. Cells in each group were seeded in 96-well plates at a concentration of 2 × 10^3^ cells per well, and left to adhere for 4 h. Then cells were incubated for 24 h, 48 or 72 h. At 0, 24, 48 and 72 h, the culture medium was discarded, and CCK-8 detection reagent was added. After incubated at 37°C for 1 h, the absorbance of cells was measured at 450 nm using a microplate reader.

### Colony-forming experiments

The cells of each group were inoculated into a 3.5 cm petri dish at a density of 1000 cells/dish. After being cultured at 37°C, 5% CO_2_ for 2 weeks, the cells were washed using PBS, and fixed with 4% paraformaldehyde for 15 min. Then the cells were stained with 0.1% crystal violet for 30 min, and observed using optical microscope, or counted using ImageJ software.

### Cell migration and Invasion assays

For transwell migration assays, cells were transferred to the top chamber of a noncoated membrane chamber in DMEM medium containing 5% fetal calf serum. And for transwell invasion assays, cells were transferred to the top chamber of a Matrigel-coated invasion chamber in DMEM medium containing 5% fetal calf serum. DMEM containing 20% fetal calf serum was added to the lower chamber to act as a chemoattractant. After incubation for 24 h, noninvasive cells were removed from the upper well, and the remaining invasive cells were fixed with 4% paraformaldehyde and stained with crystal violet. The cells were observed and photographed using an optical microscope.

### Statistical analysis description

Statistical analysis for two-group sample comparisons was performed using the Wilcoxon test, while the Kruskal-Wallis test was utilized for multiple group comparisons during statistical plotting. Significance levels were denoted as ‘ns’ (*p* > 0.05), ‘^*^’, ‘^**^’, ‘^***^’, and ‘^****^’ for *p*-values less than or equal to 0.05, 0.01, 0.001, and 0.0001, respectively.

### Data availability statement

The datasets used and/or analyzed during the current study are available from the corresponding author on reasonable request.

## Supplementary Materials

Supplementary Figures

Supplementary Table 1

Supplementary Table 2

Supplementary Table 3

Supplementary Table 4

Supplementary Table 5

Supplementary Table 6

Supplementary Table 7

Supplementary Table 8

Supplementary Table 9

Supplementary Table 10

Supplementary Tables 11 and 12
