# Supplementary material for: Single-cell RNA combined with bulk RNA analysis to explore oxidative stress and energy metabolism factors and found a new prostate cancer oncogene MXRA8
Source: Aging (Albany NY). 2024 Mar 4;16(5):4469–502. doi: 10.18632/aging.205599 (PMC10968713; doi:10.18632/aging.205599)
Supplement: Supplementary Figures [file aging-16-205599-s001.pdf]

SUPPLEMENTARY FIGURES

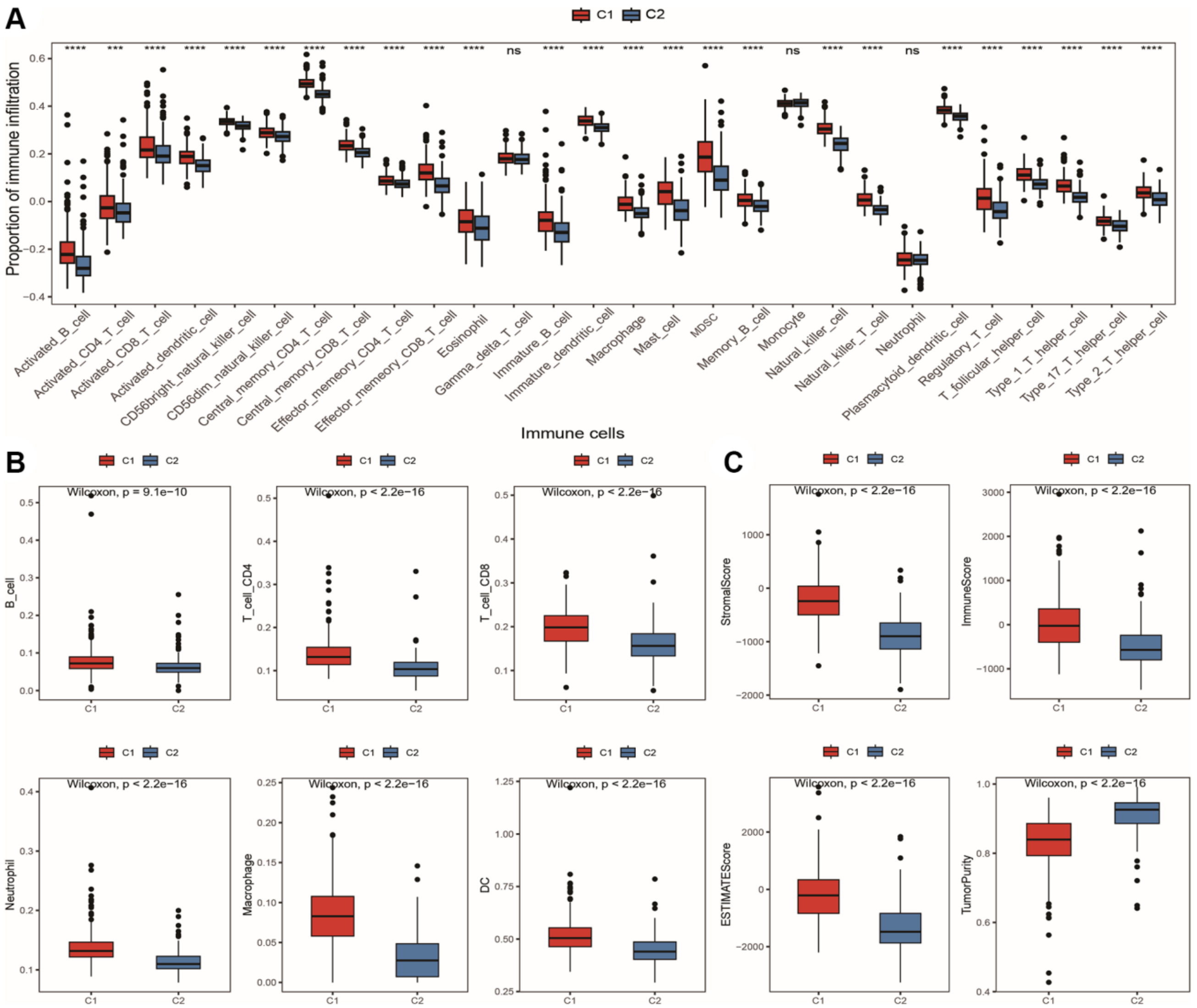

Supplementary Figure 1. Functional and immune infiltration differences in TCGA prostate cancer subtypes. (**A**) Box plot showing differences in immune cell infiltration proportions calculated by ssGSEA algorithm; (**B**) Box plot showing differences in immune cell infiltration proportions calculated by TIMER algorithm; (**C**) Box plot showing differences in stromal score, immune score, ESTIMATE score, and tumor purity, with red and blue representing C1 and C2, respectively.

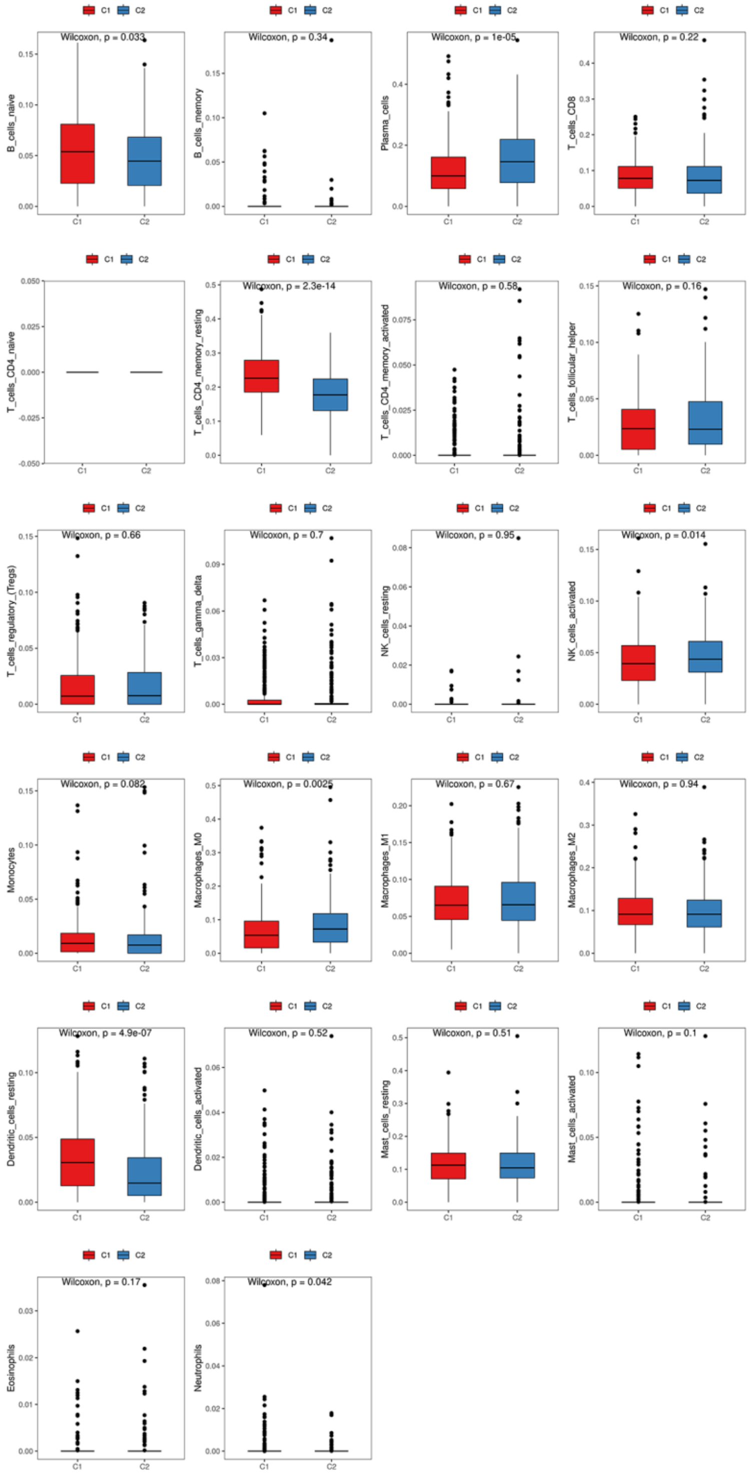

Supplementary Figure 2. Box plot showing differences in immune cell infiltration proportions calculated by CIBERSORT algorithm, with red and blue representing C1 and C2, respectively.

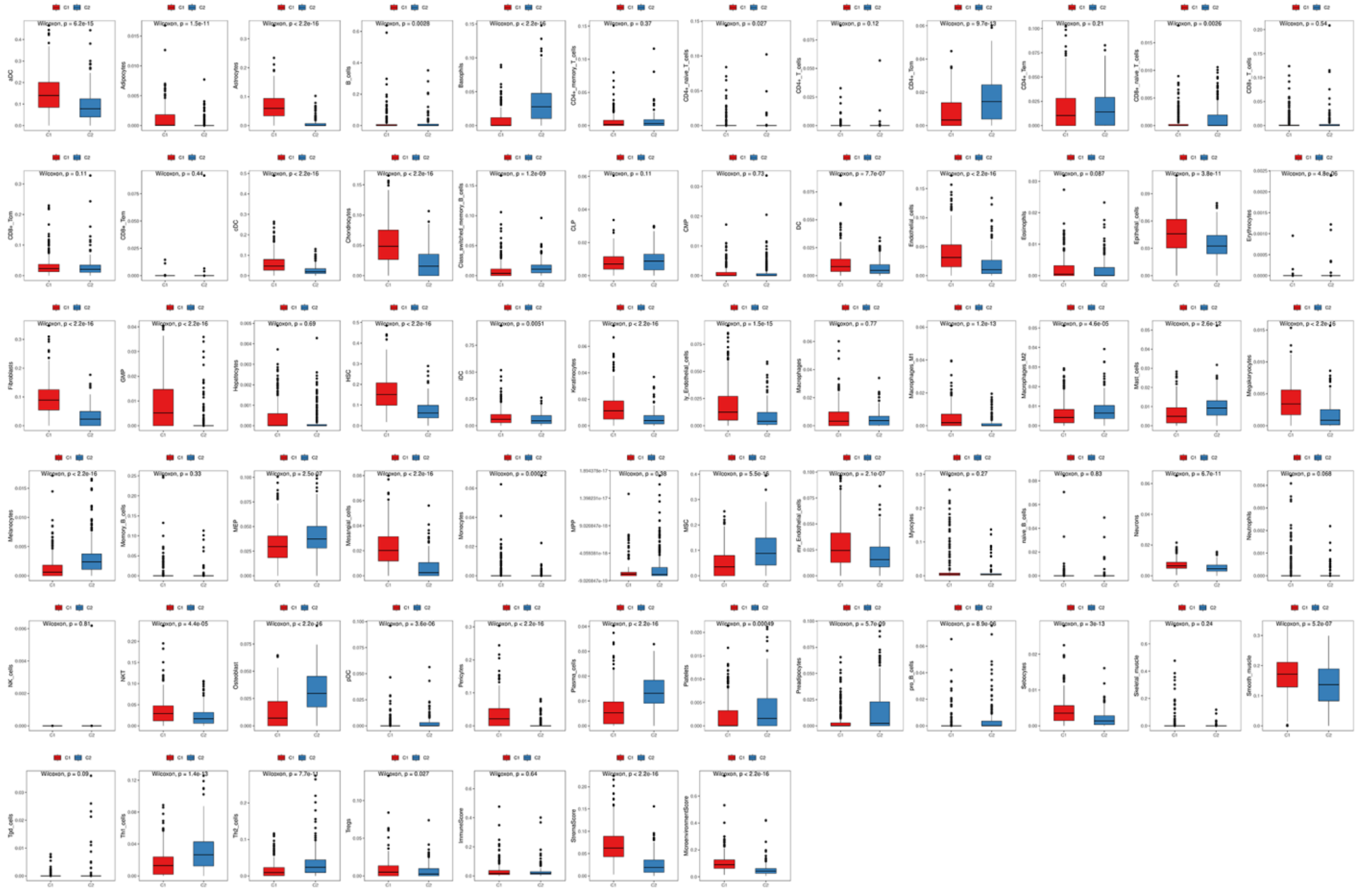

Supplementary Figure 3. Box plot showing differences in immune cell infiltration proportions calculated by xCell algorithm, with red and blue representing C1 and C2, respectively.

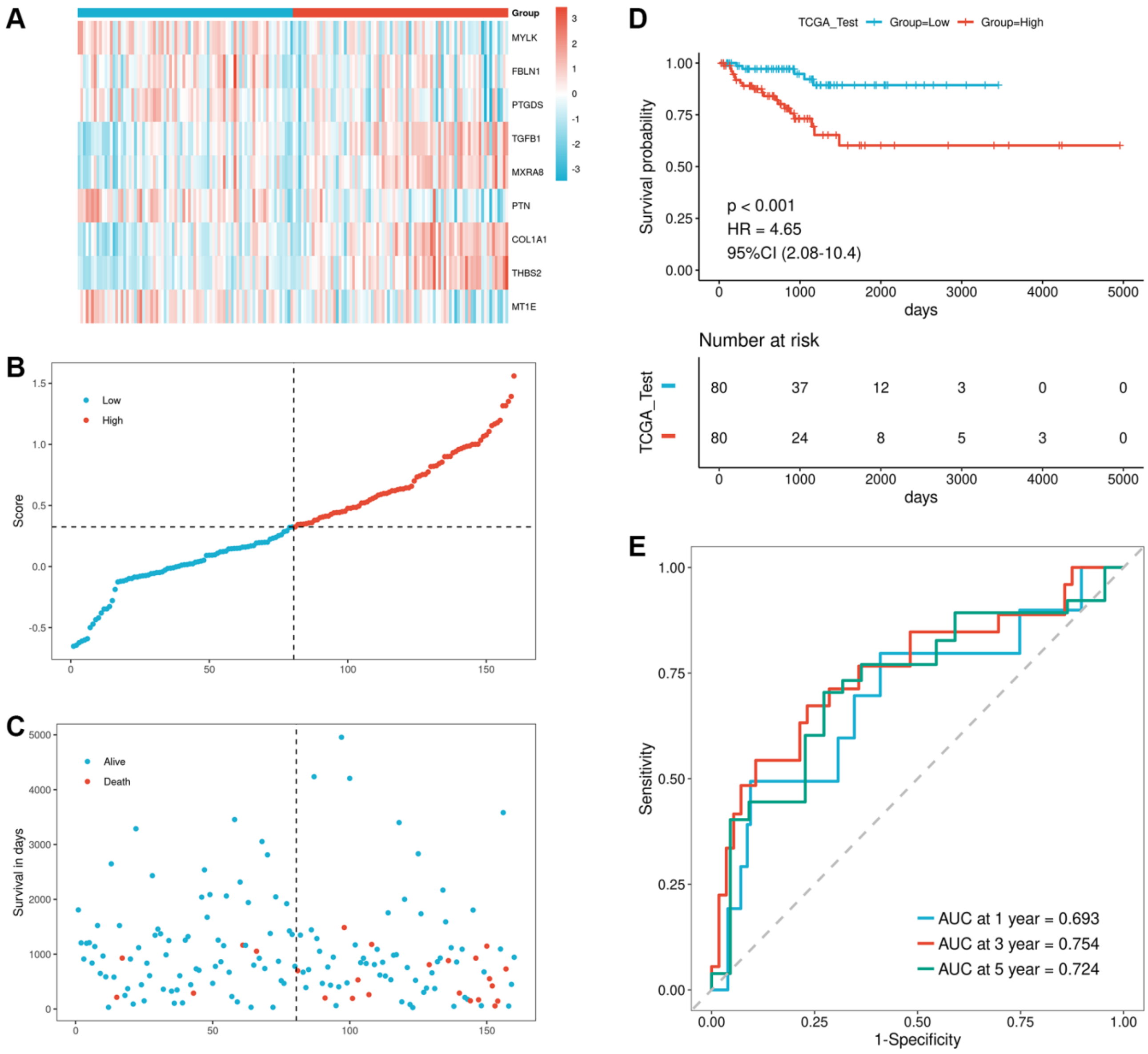

Supplementary Figure 4. Validation results of the model performance using TCGA testing set. (**A**–**C**) Risk triad plots of TCGA testing set, including heatmap of model gene expression in risk score groups, scatter plot of risk scores, and scatter plot of survival time, with red representing high-risk group and blue representing low-risk group; (**D**) KM curve of TCGA testing set; (**E**) ROC curve of the overall TCGA dataset.

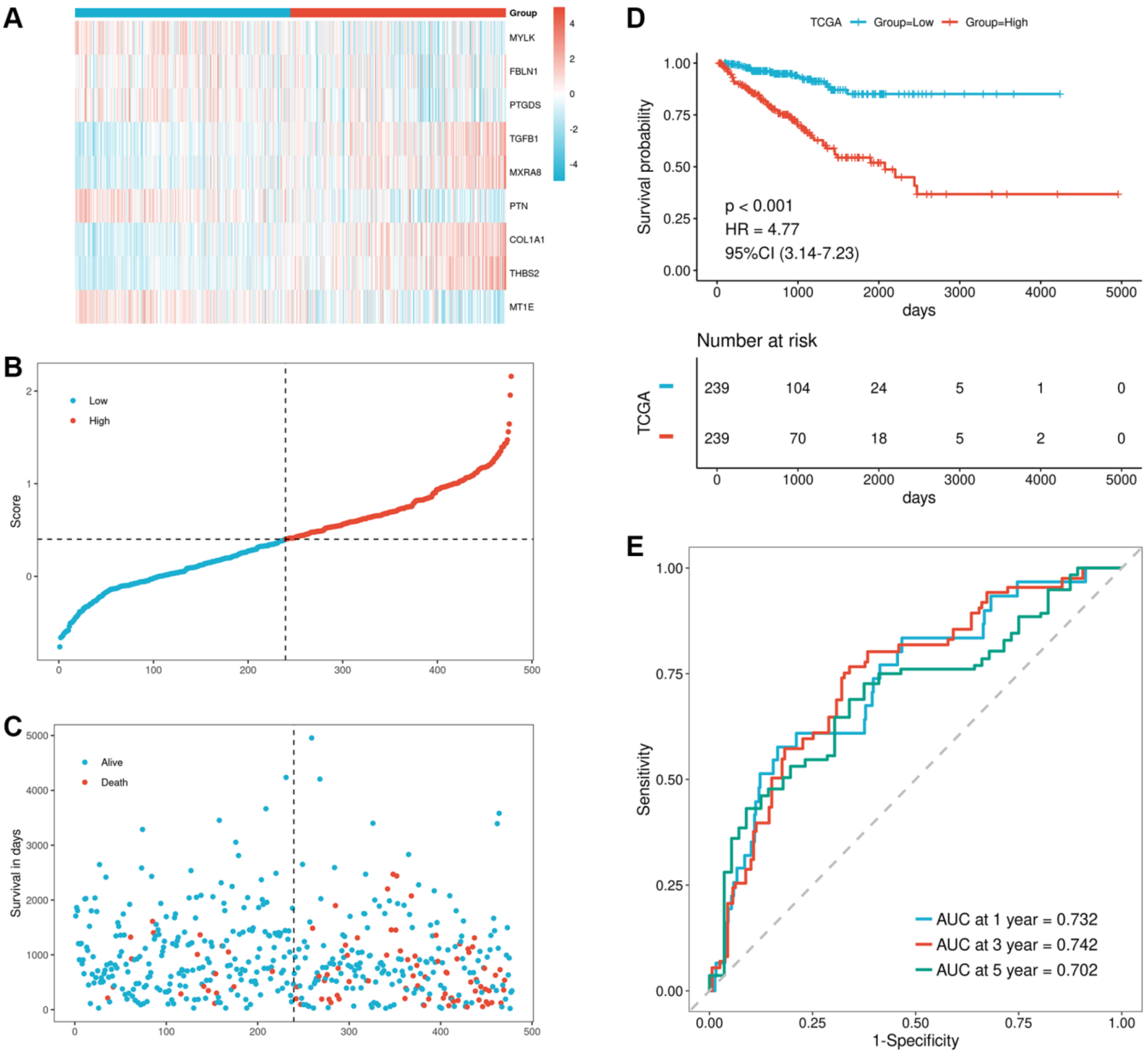

Supplementary Figure 5. Validation results of the model performance using the overall TCGA dataset. (**A**–**C**) Risk triad plots of the overall TCGA dataset, including heatmap of model gene expression in risk score groups, scatter plot of risk scores, and scatter plot of survival time, with red representing high-risk group and blue representing low-risk group; (**D**) KM curve of the overall TCGA dataset; (**E**) ROC curve of the overall TCGA dataset.

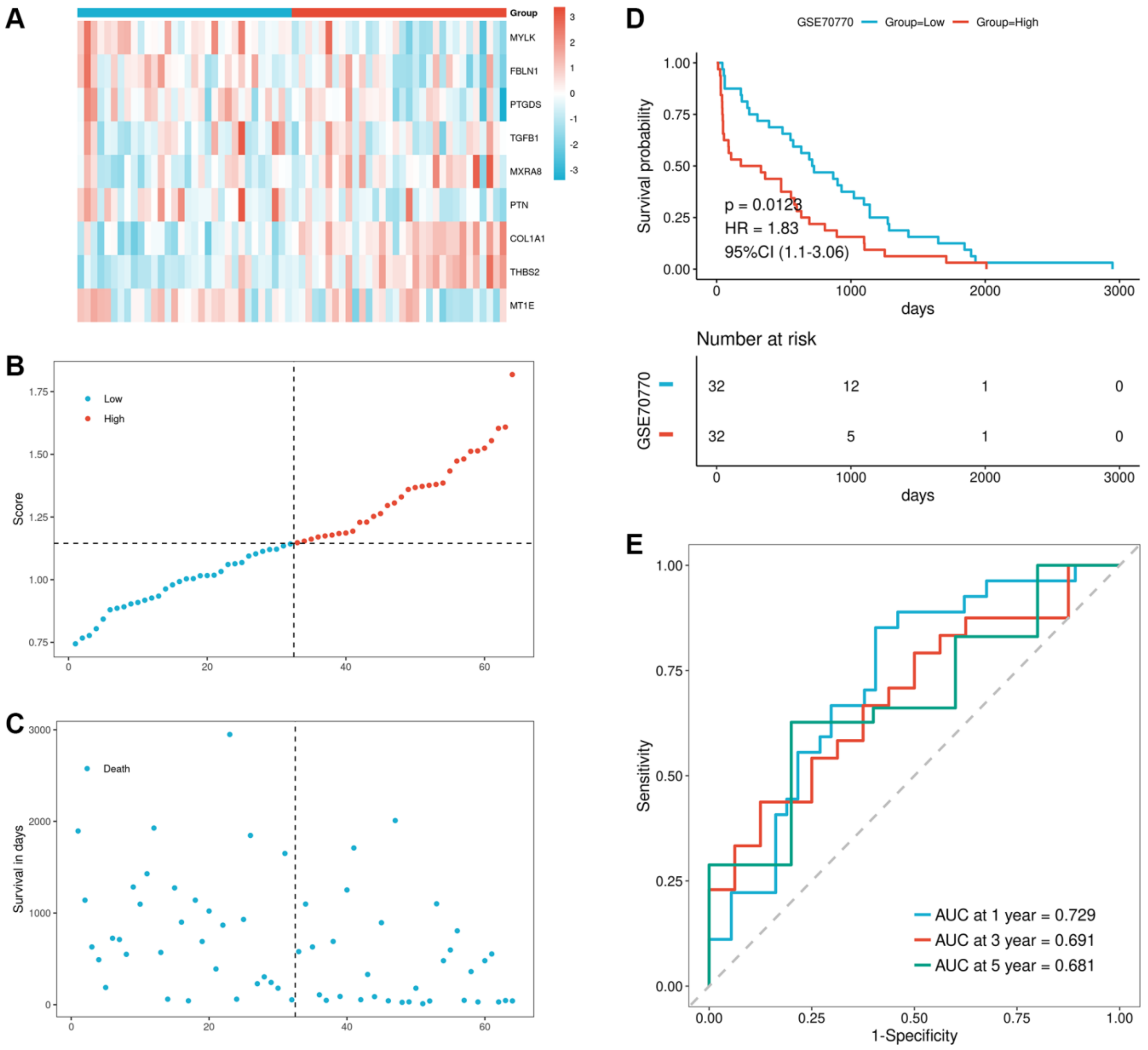

Supplementary Figure 6. Validation results of the model performance using the GSE70770 validation set. (**A**–**C**) Risk triad plots of the GSE70770 dataset, including heatmap of model gene expression in risk score groups, scatter plot of risk scores, and scatter plot of survival time, with red representing high-risk group and blue representing low-risk group; (**D**, **E**) KM and ROC curve plots of the GSE70770 dataset.

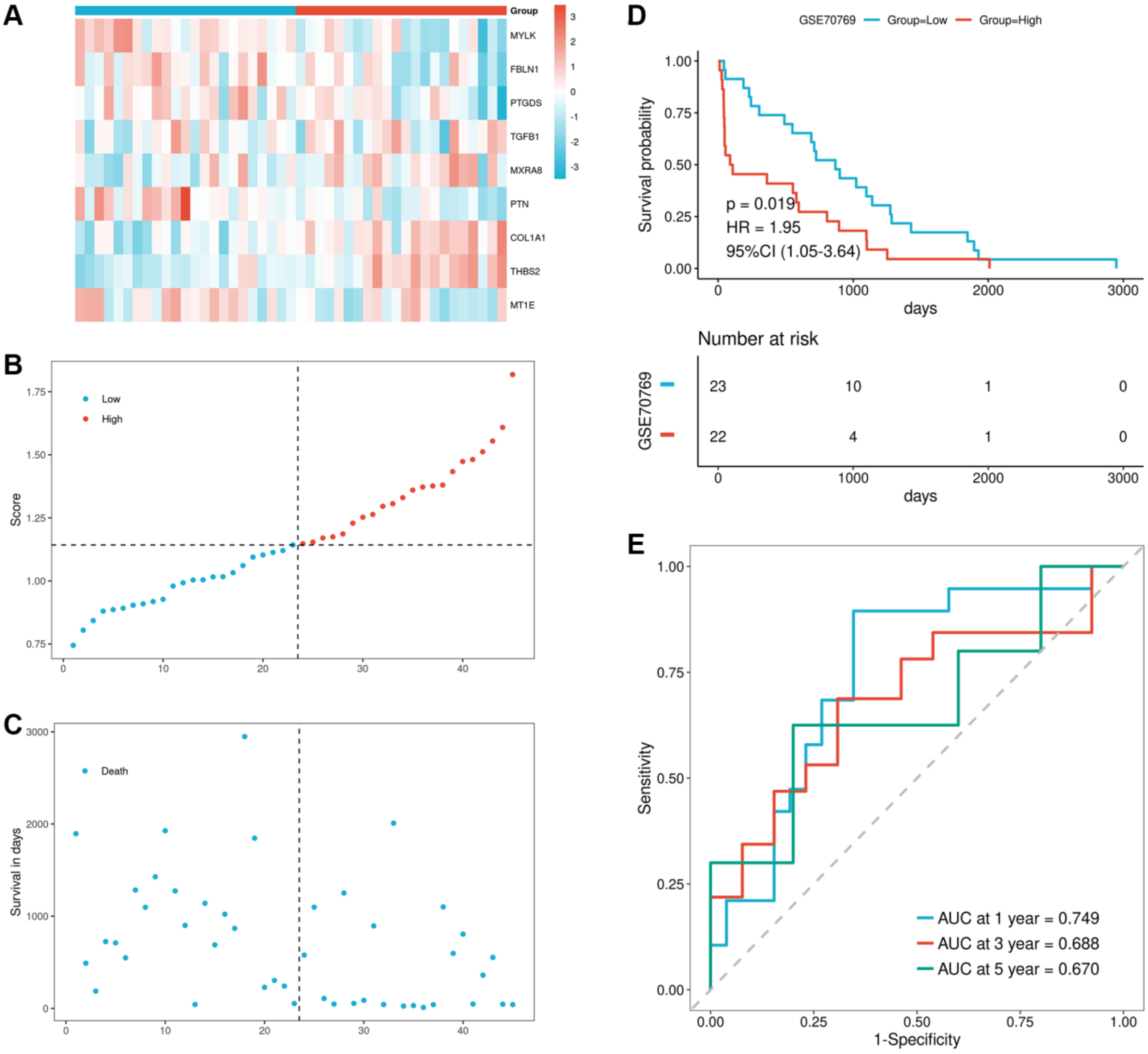

Supplementary Figure 7. Validation results of the model performance using the GSE70769 validation set. (**A**–**C**) Risk triad plots of the GSE70769 dataset, including heatmap of model gene expression in risk score groups, scatter plot of risk scores, and scatter plot of survival time, with red representing high-risk group and blue representing low-risk group; (**D**, **E**) KM and ROC curve plots of the GSE70769 dataset.

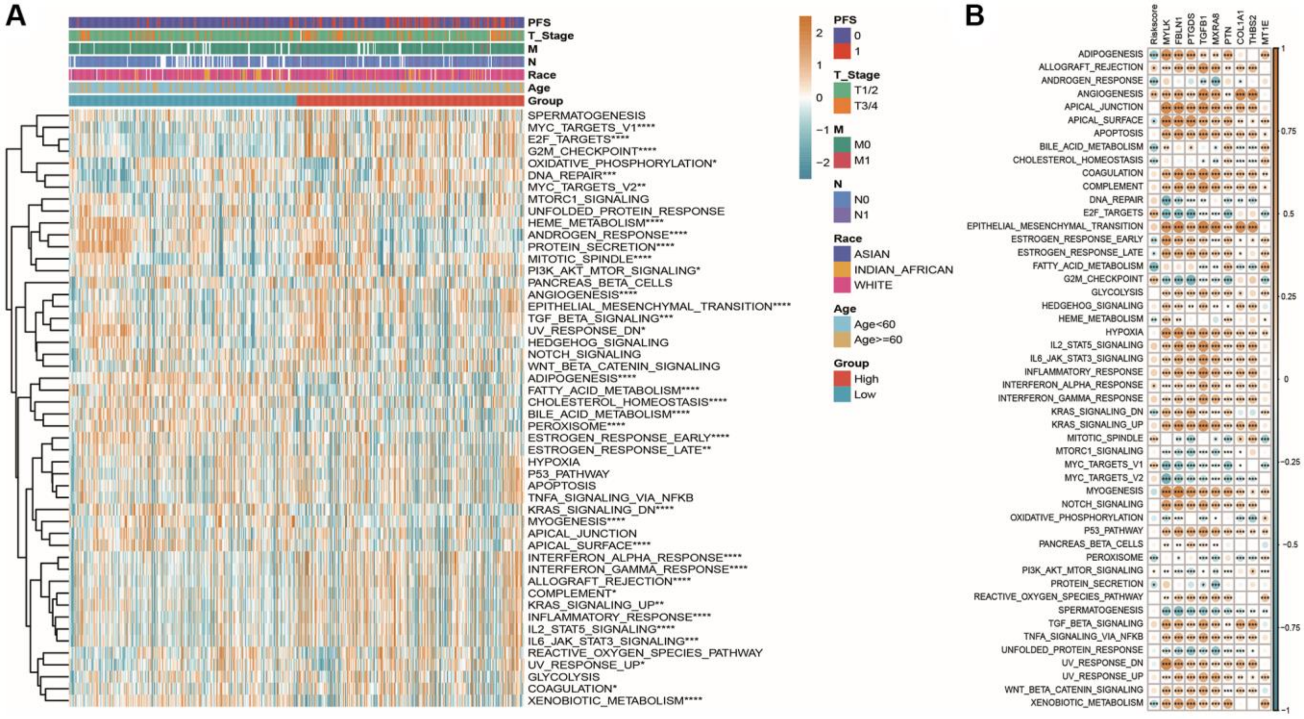

Supplementary Figure 8. Pathway enrichment differences among model groups. (**A**) Differences in HALLMARK pathway enrichment scores between high and low-risk groups, with asterisk indicating significance of differences; (**B**) Correlation heatmap between Riskscore and model gene expression with pathway enrichment scores, with color depth representing the level of correlation and *indicating significance.
