# Supplementary material for: Single-cell RNA combined with bulk RNA analysis to explore oxidative stress and energy metabolism factors and found a new prostate cancer oncogene MXRA8
Source: Aging (Albany NY). 2024 Mar 4;16(5):4469–502. doi: 10.18632/aging.205599 (PMC10968713; doi:10.18632/aging.205599)
Supplement: Supplementary Tables 11 and 12 [file aging-16-205599-s012.pdf]

SUPPLEMENTARY TABLES

Supplementary Table 11. Clinical information table of TCGA PRAD cohort.

TCGA PRAD	Group information	Number of samples
PFS	0	389
	1	89
Age	Age <60	316
	Age ≥60	162
T_Stage	T1/2	375
	T3/4	100
N	N0	335
	N1	74
M	M0	437
	M1	3
Race	ASIAN	12
	INDIAN_AFRICAN	57
	WHITE	395

Supplementary Table 12. Statistical of Oxidative Stress and Energy Metabolism Gene Set.

Related pathways	PathwayID	Gene Count
Response to oxidative stress	GO:0006979	444
Cell death in response to oxidative stress	GO:0036473	89
Cellular oxidant detoxification	GO:0098869	102
Biological oxidations	R-HSA-211859	221
Metabolism of carbohydrates	R-HSA-71387	292
Mitochondrial Fatty Acid Beta-Oxidation	R-HSA-77289	38
Glycogen synthesis	R-HSA-3322077	16
Glycogen metabolism	R-HSA-8982491	27
Glucose metabolism	R-HSA-70326	92
Glycogen breakdown glycogenolysis	R-HSA-70221	15
Glycolysis	R-HSA-70171	72
Pyruvate metabolism	R-HSA-70268	31
